# Imidazo[1,2‑*a*]pyridines as Privileged Scaffolds: Synthetic Advances, Biological Activities, and Translational Challenges

**DOI:** 10.1021/acsomega.6c07566

**Published:** 2026-07-31

**Authors:** Giorgio Antoniolli, Rebeca Chaguri, Lorraine Alves Grabauskas, Denise Costa Arruda, Tiago Rodrigues

**Affiliations:** † Institute of Chemistry, 28132 University of São Paulo, São Paulo, São Paulo 05508-000, Brazil; ‡ Center for Natural and Human Sciences, 74362 Federal University of ABC, Santo André, São Paulo 09210-170, Brazil; § Integrated Biotechnology Center, 133647 University of Mogi Das Cruzes, Mogi Das Cruzes, São Paulo 08780-911, Brazil

## Abstract

Imidazo­[1,2-*a*]­pyridine derivatives are privileged scaffolds in medicinal chemistry due to their structural versatility, favorable physicochemical properties, and broad pharmacological relevance. Their fused heterocyclic framework enables extensive functionalization while preserving conformational rigidity and optimal heteroatom distribution, making this core highly attractive for drug discovery. This systematic review summarizes recent advances (last 5 years) in the synthesis, structural diversification, and biological evaluation of imidazo­[1,2-*a*]­pyridine-based compounds. A comprehensive literature search was performed using the Scopus and Web of Science databases, focusing on studies reporting relevant biological data. The reviewed derivatives display diverse pharmacological activities, including anticancer, antimicrobial, antitubercular, antifungal, antiparasitic, neuroactive, anti-inflammatory, antidiabetic, and ferroptosis-modulating effects. Emphasis is placed on structure–activity relationships, scaffold-hopping strategies, and emerging hybrid designs. Despite promising *in vitro* potency against multiple therapeutic targets, important limitations remain. Many compounds still lack *in vivo* validation, pharmacokinetic characterization, and ADME/Tox optimization, restricting their progression toward preclinical development. In addition, structural exploration remains concentrated on conventional substitution patterns, underscoring the need for broader bioisosteric and fragment-based approaches. Overall, this review consolidates recent advances, highlights critical knowledge gaps, and outlines future directions for the rational development of imidazo­[1,2-*a*]­pyridine-based therapeutics.

## Graphical abstract

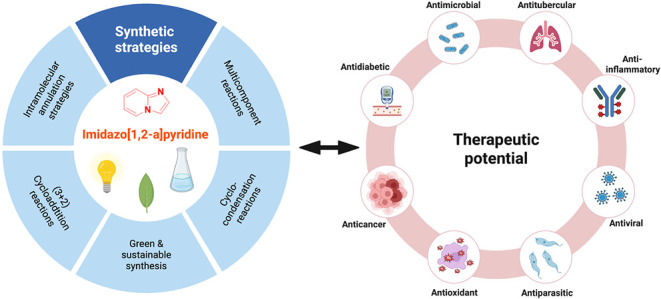

## 1 Introduction

Heterocycles are fundamental scaffolds in the design of new bioactive compounds in medicinal chemistry, owing to their structural diversity and the fact that many of them participate in a wide range of biochemical processes. Their significance arises from the extensive variability in ring size, the number and nature of the heteroatoms, which may include one or more, and the incorporation of diverse functional groups in the ring structure. Altogether, these features make heterocyclic chemistry particularly attractive. Among the most extensively investigated heterocycles, nitrogen-containing systems have gained increasing prominence in pharmaceutical research, accounting for nearly 50% of all Food and Drug Administration (FDA)-approved drugs.^1^ Notable examples include benzimidazole, benzothiazole, benzoxazole, indole, quinazolinone, among others, as well as the various forms of imidazopyridines.^2–5^ Imidazopyridines deserve special emphasis because they combine pyridine and imidazole nuclei within a single fused system. These structures may occur in several isomeric arrangements (Figure 1) and have been widely explored in the literature because of their chemical and biological relevance.^6–8^

**Figure 1:**
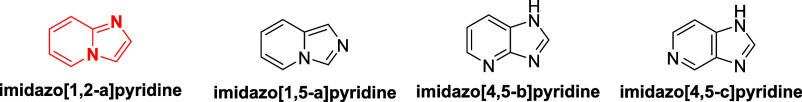
Imidazopyridine, with emphasis on isomerism on the isomer that will be discussed in this work, imidazo­[1,2-*a*]­pyridine.

The imidazo­[1,2-*a*]­pyridine nucleus is a fused heterocyclic system (5,6-bicyclic ring) that has emerged as a privileged scaffold in medicinal chemistry because of its substantial structural versatility and broad spectrum of reported bioactivities.^9–11^ This versatility is reflected not only in research compounds but also in clinically approved drugs, including sedatives and other therapeutic agents based on the imidazo­[1,2-*a*]­pyridine framework^12–15^ (Figure 2). Moreover, the fused heterocyclic architecture provides conformational rigidity, an advantageous density of heteroatoms (particularly nitrogen atoms), and flexibility for chemical modifications at multiple positions of the ring. These features facilitate the exploration of specific molecular interactions and optimization of key pharmacological properties, such as affinity, selectivity, and pharmacokinetic behavior. Given the global challenges posed by diseases such as cancer, infections, and neurodegenerative disorders, as well as the ongoing need for therapies with improved efficacy and safety profiles, versatile scaffolds such as imidazo­[1,2-*a*]­pyridine remain highly valuable in contemporary drug discovery and development research.

**Figure 2:**
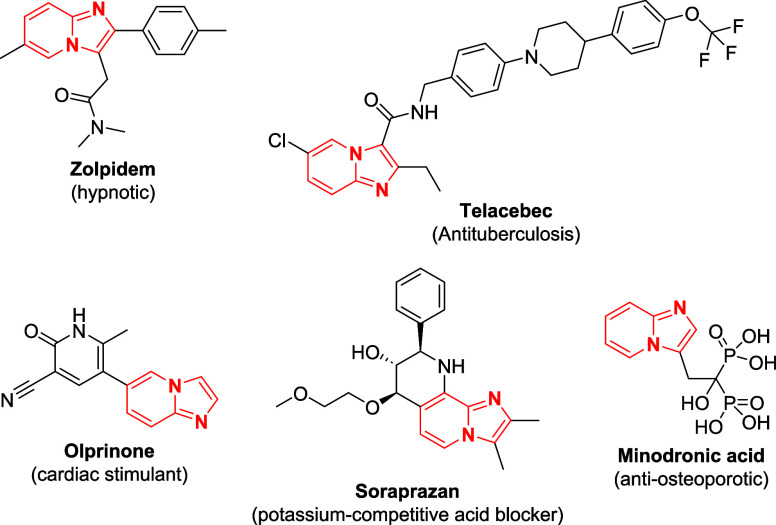
Drugs featuring imidazo­[1,2-*a*]­pyridine scaffolds have a wide range of pharmacological applications. Although not all of these drugs are approved by the FDA, zolpidem remains the most widely used and clinically established imidazo­[1,2-*a*]­pyridine-derived drug.

Although historical reviews have addressed imidazo­[1,2-*a*]­pyridine as a promising scaffold, substantial recent progress, encompassing new synthetic methodologies, emerging therapeutic applications, and expanded derivative classes, warrants a critical update. In this regard, more efficient and sustainable synthetic approaches have been reported, including visible-light-induced C–H functionalization reactions.^16^ Moreover, imidazo­[1,2-*a*]­pyridine derivatives continue to emerge as promising candidates for multiple therapeutic targets, including anticancer and antitubercular applications, for which focused reviews have been published recently.^17,18^ In other words, there is a clear need to consolidate these recent findings, including how frequently the imidazo­[1,2-*a*]­pyridine scaffold has been leveraged in scaffold-hopping strategies, which subfamilies have emerged as particularly relevant, which therapeutic targets have been most extensively investigated, and which challenges remain, such as solubility, toxicity, and selectivity. Collectively, these aspects underscore the importance of providing an updated systematic overview.

A historical overview, together with recent trends, highlights the continuous pursuit of new synthetic methodologies for imidazo­[1,2-*a*]­pyridines (Figure 3). Traditionally, most of these derivatives have been prepared by reacting 2-aminopyridine with an α-halocarbonyl compound, a transformation that can be carried out under relatively mild conditions.^19,20^ A recent study reported an innovative and straightforward method for synthesizing imidazo­[1,2-*a*]­pyridines via a NaIO_4_ (sodium metaperiodate)/*tert*-butyl hydroperoxide (TBHP)-promoted (3 + 2) cycloaddition reaction between propargyl alcohols and 2-aminopyridines. This strategy exhibited broad functional group tolerance and enabled the generation of a diverse set of C-3-carbonylated imidazo­[1,2-*a*]­pyridines.^21^ Another noteworthy method employed one-pot synthesis to convert β-O-4 model compounds, which are representative segments of lignin, into imidazo­[1,2-*a*]­pyridines with yields of up to 95%, using 2-aminopyridine as the nitrogen source.^22^ An eco-friendly and metal-free method for synthesizing 3-aminoimidazo­[1,2-*a*]­pyridines was developed through a multicomponent reaction involving benzylamine, 2-aminopyridine, and *tert*-butyl isocyanide under visible-light irradiation. In this protocol, eosin Y serves as a photoredox catalyst, enabling mild reaction conditions, one-pot operation, and broad tolerance to both electron-donating and electron-withdrawing substituents. These features provide significant advantages in terms of sustainability and synthetic scope.^23^

**Figure 3:**
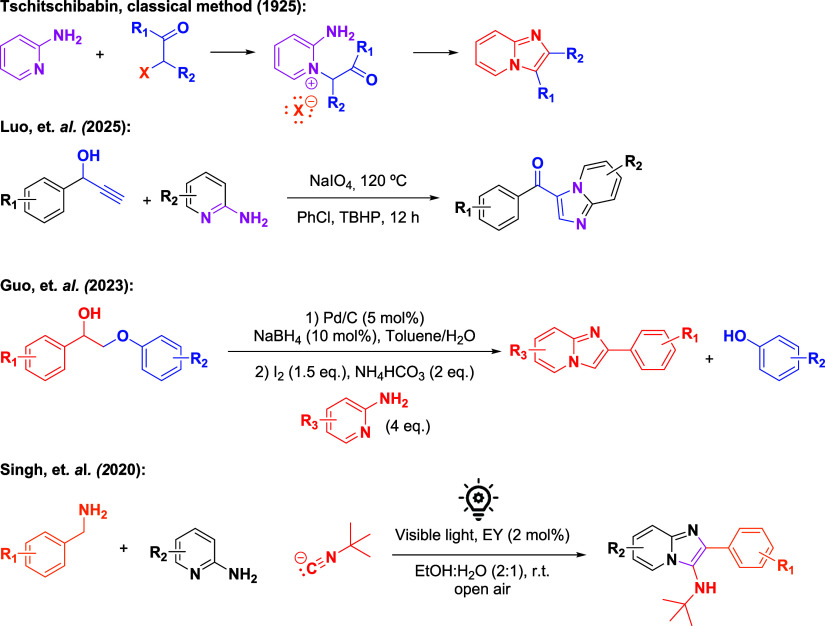
A few representative synthetic methods for obtaining imidazo­[1,2-*a*]­pyridines are briefly presented for illustrative purposes, as the primary aim of this paper is to address the biological activities of this class of heterocycles. TBHP: Tert-butyl hydroperoxide; EY: Eosin Y.

Imidazo­[1,2-*a*]­pyridines have a wide variety of biological activities, such as anticancer,^24–32^ antimicrobial,^33–36^ antiviral,^37–39^ anti-inflammatory,^40–42^ neurological activity,^43–47^ antidiabetic,^48,49^ antitubercular,^50–53^ antifungal,^54,55^ anticonvulsant,^56,57^ antioxidant,^58^ antikinetoplastid,^59^ and antiparasitic.^60,61^ Therefore, this review, following the proposed methodology (a systematic search of the Scopus and Web of Science databases), aims to provide an up-to-date overview (past 5 years) of the state of the art, including structural modifications, biological activities, emerging trends, and future perspectives. Within this framework, several objectives guide the scope of this review: (i) to identify and compile recent advances in the structural modification of imidazo­[1,2-*a*]­pyridine derivatives; (ii) to assess and summarize their biological profiles, with emphasis on the most extensively explored therapeutic classes, such as anticancer, antifungal, antitubercular, and antikinetoplastid activities, as well as additional applications including anti-Alzheimer and anti-inflammatory effects; and (iii) to discuss scaffold-hopping strategies, current limitations, and potential research gaps that may inform future investigations.

## 2 Methods

In this review, we systematically identify, analyze, and summarize recent advances in the discovery of new molecules incorporating the imidazo­[1,2-*a*]­pyridine nucleus, a well-recognized privileged scaffold and established pharmacophore in medicinal chemistry. To this end, we conducted a comprehensive online search on the Scopus and Web of Science platforms using the keywords “imidazo­[1,2-*a*]­pyridine,” “synthesis,” and “biological evaluation”. The search covered publications from 2026 to 2021, and only studies reporting relevant biological data and published in high-impact journals were included.^62,63^ The retrieved articles were organized according to the range of biological activities reported for the imidazo­[1,2-*a*]­pyridine derivatives. These activities encompass a broad pharmacological spectrum, including anticancer, antituberculosis, anti-Alzheimer’s disease, antifungal, G6PD inhibition, and ferroptosis modulation. This approach provides a structured and comprehensive overview of how this privileged scaffold has been explored in multiple therapeutic areas in recent years. The graphical abstract was prepared using the BioRender platform under an Academic Publication License. It is important to emphasize that no form of artificial intelligence was used in the preparation of this manuscript, including the generation of text and figures. However, the artificial intelligence platform Paperpal was used to proofread and refine the text.

## 3 Investigation of Imidazo[1,2-*a*]pyridine as Pharmacological Agents

Despite the growing number of applications of imidazo­[1,2-*a*]­pyridines, several gaps remain. Structural diversity is still largely confined to specific positions or substitution patterns, indicating substantial scope for exploring new substitution motifs, linkers, hybrid frameworks, and bioisosteric strategies. Another relevant point is that many reported compounds exhibit promising *in vitro* activity but lack advanced pharmacokinetic, *in vivo* toxicity, selectivity, and bioavailability data, which hinders their progression toward preclinical candidates. Most studies focus primarily on potency and cellular assays; however, deeper investigations, such as ADME/Tox modeling and optimization are still scarce. In this context, this updated systematic review documents recent advances, current challenges, and potential research gaps, providing a strategic foundation for the future development of imidazo­[1,2-*a*]­pyridine-based compounds.

### 3.1 Anticancer

Geng and coworkers investigated 3-(5-(butylthio)-1H-1,2,4-triazol-3-yl)-2-ethylimidazo­[1,2-*a*]­pyridine as a potential anticancer agent (Figure 4). This compound was synthesized and its structure optimized through Density Functional Theory (DFT) calculations, which were subsequently compared with the structure obtained from X-ray diffraction analysis. Notably, the HOMO–LUMO energy gap of 4.47 eV indicated a moderate tendency toward chemical reactivity. Compound **1** exhibited an IC_50_ (half maximal inhibitory concentration) value of 39.65 μM against the MV-4–11 acute myeloid leukemia cell line, whereas the reference drug doxorubicin showed an IC_50_ of 0.06 μM under the same conditions. In contrast, compound **1** displayed no significant activity against the HT-29 (colorectal cancer) cell line (IC_50_ > 50 μM). No structure–activity relationship (SAR) studies were conducted in this study because only compound 1 was evaluated for antiproliferative activity. Molecular docking studies revealed a binding energy of −24.7 kcal·mol^–1^ with the 1WT5 protein (PDB code), suggesting strong affinity toward the target. The compound established hydrogen-bond interactions with residues Gln39 (1.82 Å), Tyr92 (3.05 Å), and Ala9 (2.20 Å and 2.74 Å), in addition to several π–alkyl interactions within the active site. These findings suggest that this molecule may serve as a promising lead for further optimization to enhance its antitumor potency.^64^

**Figure 4:**
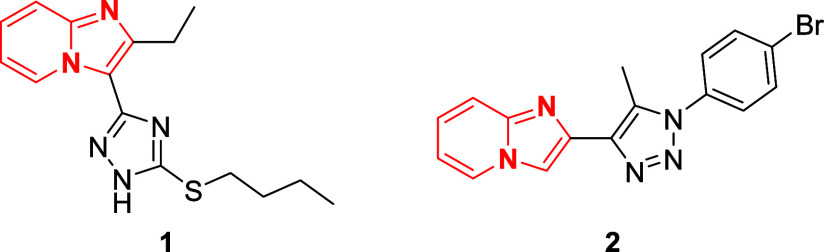
Structures of compounds **1** and **2** exhibiting anticancer activity.

Cojocaru and colleagues conducted theoretical investigations on compound **2**, comparing the computational results with available experimental data from X-ray crystallography and spectroscopic analyses (Figure 4). Notably, the electrostatic potential map of **2** revealed potential electrophilic attack sites at the nitrogen atom of the imidazole ring and the nitrogen atoms within the 1,2,3-triazole scaffold. Given that compound **2** contains hydrophobic moieties and is practically insoluble in water, increasing its polarity enhances its ability to form hydrogen bonds. This study evaluated only this compound. The HOMO–LUMO energy gap was calculated to be 4.742 eV according to DFT analysis, suggesting that the molecule is relatively rigid and electronically less polarizable. The study further demonstrated that compound **2** complies with all five commonly used drug-likeness filters, Lipinski, Ghose, Veber, Egan, and Muegge, indicating favorable oral bioavailability potential and overall drug-like properties. An *in silico* anticancer investigation was performed to evaluate the potential of **2** as an inhibitor of epidermal growth factor receptor (EGFR) tyrosine kinase. The PDB code 3POZ was employed using the ligand in its protonated form (**2**-H^+^). The protonated **2** ligand (guest) was docked into β-cyclodextrin (β-CD, host) to explore its potential as an optimized therapeutic candidate and subsequently docked into EGFR as a target receptor. The binding affinity of the β-CD@**2**-H^+^ complex was calculated to be −5.92 kcal·mol^–1^, whereas that of the EGFR@**2**-H^+^ complex was −8.77 kcal·mol^–1^, with a dissociation constant (*K*_*d*_) of 0.372 μM. Furthermore, a 20 ns molecular dynamics simulation confirmed the structural stability of the EGFR@**2**-H^+^ complex throughout the simulation.^65^

Peixoto and coworkers investigated a series of imidazo­[1,2-*a*]­pyridine derivatives to identify potent and selective inhibitors of salt-inducible kinase 1 (SIK1). The SIK family comprises three isoforms, SIK1, SIK2, and SIK3, which are members of the AMP-activated protein kinase (AMPK) serine/threonine kinase family. SAR studies identified a specific substitution pattern on the phenyl ring that enhanced potency against the SIK1 isoform, where the 4-chloro-2,6-dimethyl substituents were opposite the previous ones, and selectivity over SIK2 and SIK3. Through structural optimization, the group developed compound **3**, a subnanomolar SIK1 inhibitor in biochemical assays, which displayed over 100-fold selectivity against SIK2 and SIK3 (Figure 5). In a nanobioluminescence resonance energy transfer (NanoBRET) binding assay, compound **3** exhibited even higher selectivity, over 400-fold, for SIK1 relative to SIK2 and SIK3. Furthermore, compound **3** was evaluated for the percent inhibition of enzymatic activity at a concentration of 1 μM across a panel of 392 kinases. The compound demonstrated excellent selectivity within the CMAK kinase group and strong inhibition of multiple tyrosine kinases. Compound **3** exhibited potent activity (IC_50_ ≤ 10 nM) against 15 tyrosine kinases, including members of the SRC family (FGR, HCK, LYN, YES1, LCK, FYN, SRC, and FRK), which are well-established cancer targets and are inhibited by several clinically approved tyrosine kinase inhibitors. All findings reported in this study warrant further investigation, and off-target activity toward tyrosine kinases should not be overlooked in the interpretation of biological data.^66^

**Figure 5:**
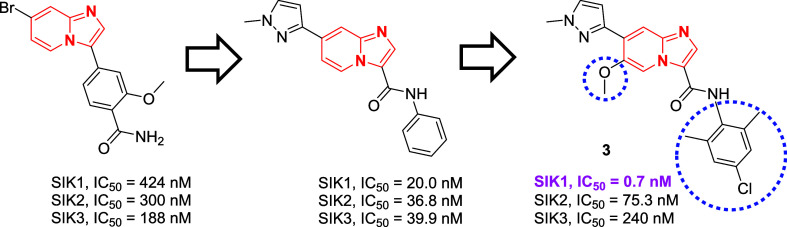
Progression of structure–activity relationship studies leading to the development of compound **3**, a potent SIK1 inhibitor.

Dash and collaborators synthesized and evaluated noscapine derivatives, also known as narcotine, an alkaloid, against two breast adenocarcinoma cell lines (MCF-7 and MDA-MB-231). Four derivatives were obtained, exhibiting antiproliferative activities below 25.2 μM for MCF-7 and below 32.4 μM for MDA-MB-231 (Figure 6). The most active compound, **4**, bearing a 6-CH_3_ substituent, exhibited IC_50_ values of 3.7 and 5.6 μM for MCF-7 and MDA-MB-231 cells, respectively. The reference compound, noscapine, exhibited IC_50_ values of 44.1 and 57.4 μM for MCF-7 and MDA-MB-231 cells, respectively, demonstrating the superior potency of compound **4**. None of the compounds exhibited cytotoxic activity against the human embryonic kidney (HEK) cells (IC_50_ > 300 μM). Interestingly, variations in the substituents at positions 6 and 5 led to changes in the antiproliferative activity. For both cancer cell lines, the anticancer potency was in the following descending order: 6-CH_3_ (**4**) > 6-I > 5-Br ≫ 5-Cl ≫ noscapine. Flow cytometry analysis revealed that treatment with compound **4** resulted in a higher percentage of cells arrested in the G_2_/M phase and induced apoptosis. Furthermore, this derivative significantly reduced tumor volume in an *in vivo* MCF-7 xenograft mouse model compared to the untreated group, without causing any detectable toxic effects in vital organs.^67^

**Figure 6:**
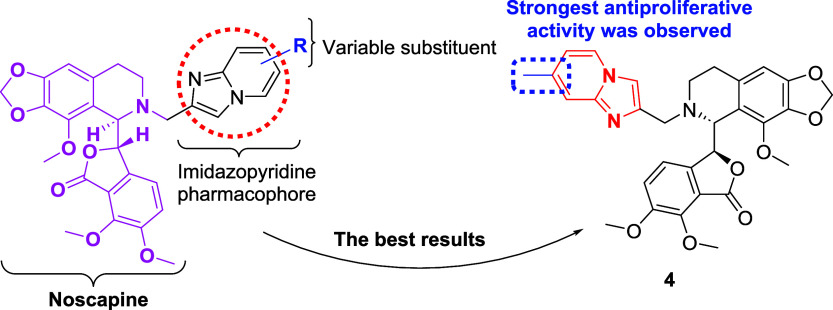
Noscapine derivatization led to the formation of compound **4**, which exhibited antiproliferative activity against MCF-7 and MDA-MB-231 cell lines.

A study conducted by Horai and colleagues identified a potent, orally bioavailable imidazopyridine derivative as a potential inhibitor of BRD4 (bromodomain-containing protein 4). Their working hypothesis was that mitigating P-glycoprotein (P-gp) substrate recognition could enhance cellular permeability, and thereby improving intracellular inhibitory activity. To test this hypothesis, the authors reduced the number of hydrogen-bond donors through *N*-alkylation. Although this strategy successfully increased cell-based inhibitory potency by attenuating P-gp-mediated efflux, the resulting compounds exhibited poor metabolic stability. To overcome this limitation, researchers discovered imidazopyridine derivative **5** (Figure 7). This compound exhibited potent inhibitory activity against BRD4-BD1 (the first bromodomain), with an IC_50_ value of 1.8 nM. Compound **5** also exhibited strong cellular inhibition of BRD4 (IC_50_ = 7.7 nM) and potent antiproliferative activity against the human malignant melanoma cell line A375.S2 (IC_50_ = 7.8 nM). Notably, the incorporation of a 1,3-dimethylpyridonyl moiety, previously associated with enhanced potency, was retained and contributed to a favorable activity profile. It is also important to note that replacing the benzimidazole ring with an imidazo­[1,2-*a*]­pyridine ring significantly improved the biological activities. **5** demonstrated good microsomal stability in both human and mouse systems (HLM/MLM CL_int_ = 10/21 μL·min^–1^·mg^–1^ protein). Unfortunately, the lead compound exhibited poor tolerability in A375.S2 cells. Xenograft-bearing mice, prompting the discontinuation of further *in vivo* evaluation. Overall, **5** exhibited potent antiproliferative activity in melanoma cells and favorable oral exposure. These findings reinforce the notion that mitigating P-gp substrate recognition can effectively enhance cellular activity and support the identification of highly promising antitumor agents.^68^

**Figure 7:**
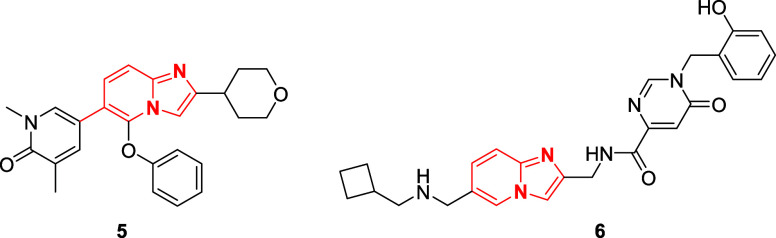
Structures of compounds **5** and **6**, which exhibit potent antiproliferative activity.

Miao and colleagues identified a series of 2,6-disubstituted imidazo­[1,2-*a*]­pyridine derivatives as potent inhibitors of METTL3 (methyltransferase-like protein 3). Among the thirty-one designed and synthesized molecules, compound **6** emerged as the lead candidate (Figure 7). Compound **6** displayed an IC_50_ value of 45.31 nM against METTL3, compared to the reference inhibitor STM2457, which showed an IC_50_ of 21.12 nM. Although one derivative exhibited slightly stronger METTL3 inhibition than **6**, its antiproliferative activity was markedly inferior, supporting **6** as the optimal candidate. SAR analysis revealed the following descending trend in METTL3 inhibition: STM2457 ≈ 2-fluoro-6-methylbenzyl > 2-fluorobenzyl > 2-hydroxybenzyl (**6**) > 3-methylbenzyl > 3-trifluoromethylbenzyl > all other derivatives (IC_50_ > 100 nM). When evaluated against SKOV3 (ovarian cancer) and MOLM-13 (acute myeloid leukemia) cell lines, STM2457 exhibit IC_50_ values of 5.41 and 12.03 μM, respectively. The 3-methylbenzyl analog showed slightly improved potency in SKOV3 cells (IC_50_ = 5.22 μM) but performed worse than **6** in MOLM-13 cells. In contrast, **6** exhibited IC_50_ values of 6.42 μM (SKOV3) and 12.34 μM (MOLM-13), closely matching those of the positive control. Both **6** and STM2457 demonstrated low cytotoxicity toward LO2 normal human hepatocytes. Molecular docking analysis revealed that the binding mode of **6** overlapped with that of STM2457 within METTL3. Compound **6** preserved the key hydrogen bonds with Ile378, Asn549, and Asp385 and formed a hydrogen bond with Thr408 via its phenolic hydroxyl group. To assess whether **6** modulates global m^6^A (*N* ^6^-methyladenosine) levels, *dot blot* assays were performed using SKOV3 and MOLM-13 cells. The **6**-treatments resulted in a dose-dependent reduction in total m^6^A levels in both cell lines. Furthermore, **6** induced apoptosis, inhibited cell migration, and downregulated the expression of c-MYC (cellular MYC) and BCL2 (B-cell lymphoma 2), while also demonstrating favorable pharmacokinetic properties. In an *in vivo* ovarian cancer xenograft model, treatment with **6** at 50 mg·kg^–1^ significantly suppressed tumor growth, exhibiting efficacy comparable to that of STM2457.^69^

Siddappa and colleagues discovered that 2-pyrazolines inhibit the phosphorylation of signal transducer and activator of transcription 3 (STAT3), acting as cytotoxic agents in the nanomolar range. In a previous study, the group reported the synthesis of ITPs (imidazo­[1,2-*a*]­pyridine-tethered pyrazolines), which inhibited STAT3 phosphorylation in estrogen receptor-positive (ER+) breast cancer cells, specifically MCF-7, and blocked STAT3 nuclear translocation. In the present work, they developed a new library of 2-pyrazolines linked to the imidazo­[1,2-*a*]­pyridine scaffold and their corresponding amide derivatives. Among the synthesized and evaluated compounds, compounds **7** and **8** exhibited potent cytotoxic activity against MCF-7 breast cancer cells, with IC_50_ values of 55 nM and 15 nM, respectively (Figure 8). Under the same experimental conditions, the positive controls tamoxifen and doxorubicin reduced MCF-7 cell viability with IC_50_ values of 1.75 μM and 0.73 μM, respectively. Notably, no other compound in the series exhibited comparable potency; the next most active compound had an IC_50_ of 3.65 μM, making **7** and **8** 66-fold and 243-fold more potent, respectively. Compound **8** was further evaluated in the nontumorigenic MCF-10A cell line, where it exhibited an IC_50_ of 125.1 μM, indicating low cytotoxicity toward nontransformed cells. Molecular docking studies using the *R*-enantiomer of **8** with the SH2 domain of STAT3 showed a calculated binding energy of −9.56 kcal·mol^–1^, compared to −9.27 kcal·mol^–1^ for the previously reported ITP. The nitrogen atom of the imidazo­[1,2-*a*]­pyridine ring formed two hydrogen bonds with Arg609 at distances below 2.2 Å. Compound **8** was also evaluated for its effects on pSTAT-Tyr705 levels in MCF-7 cells, and treatment with this compound resulted in significantly reduced pSTAT-Tyr705 levels relative to those in vehicle-treated controls. Furthermore, in a live/dead cell assay, **8** induced dose-dependent cell death in MCF-7 cells, with up to 80% of cells classified as dead following treatment with 5 μM of the lead compound. Overall, this study identified **8** as a highly promising STAT3 inhibitor, with strong potential for further structural optimization and future *in vivo* evaluation for oncological applications.^70^

**Figure 8:**
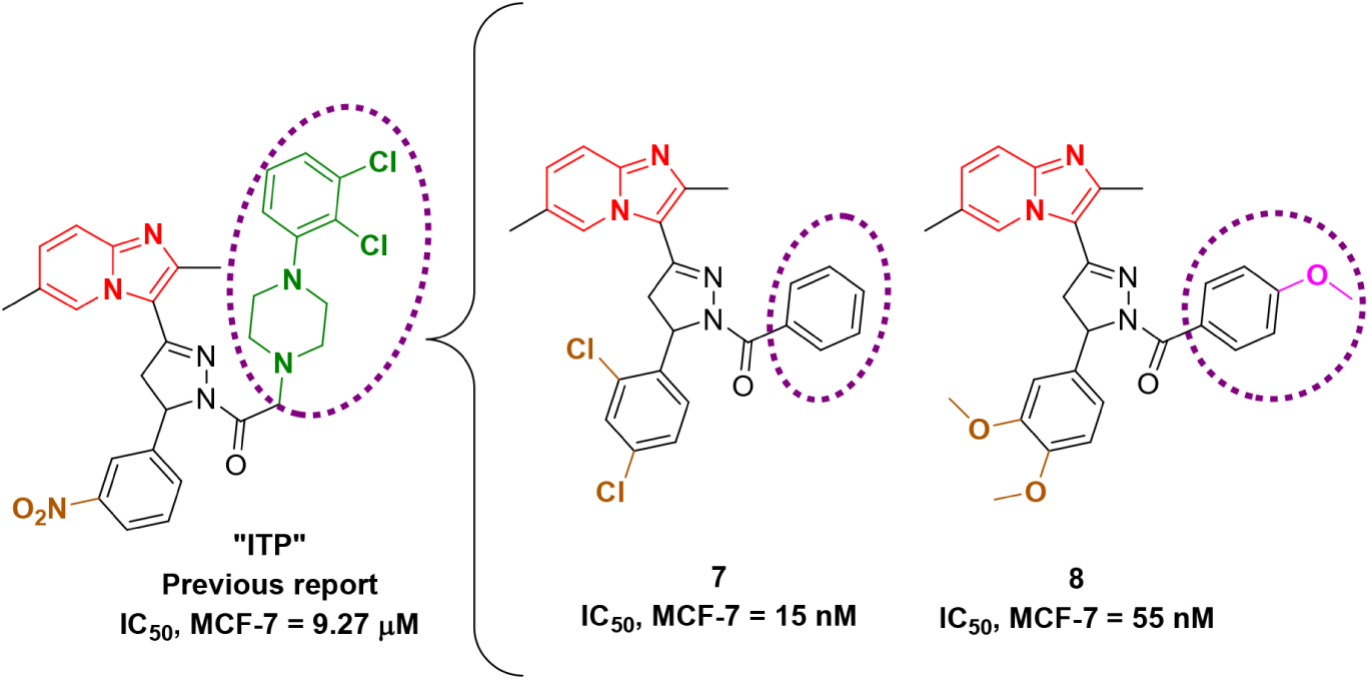
Structural differentiation between precursor ITP, reported in a previous study, and derived compounds **7** and **8**, which exhibited markedly enhanced antiproliferative activities.

An insightful study explored novel 4-(imidazo­[1,2-*a*]­pyridine-3-carbonyl)­phenylurea derivatives as potent FLT3 (fms-like tyrosine kinase 3) inhibitors using a machine-learning-guided design strategy. Several novel imidazo­[1,2-*a*]­pyridine-based compounds were synthesized to inhibit FLT3 and its downstream signaling proteins, phosphorylated protein kinase B (p-AKT) and phosphorylated extracellular signal-regulated kinases 1 and 2 (p-ERK1/2). Huang and colleagues identified the lead compound **9**, which displayed strong selectivity and bioactivity against FLT3-ITD (internal tandem duplication), with an IC_50_ of 1.4 nM, and against the acute myeloid leukemia cell line MV4–11, with an IC_50_ of 15 nM. For comparison, the positive control, quizartinib, exhibited IC_50_ values of 2.4 and 9 nM, respectively (Figure 9). Interestingly, no clear correlation was observed between FLT3 inhibition and anti-MV4–11 activity in the compound series. A comparison between 6-COOCH_3_ and 6-COOH analogs revealed substantial differences in activity against MV4–11 cells, indicating that cellular permeability likely plays a key role in the discrepancy between FLT3-ITD inhibition and MV4–11 cytotoxicity. The introduction of substituents such as 6-COOCH_3_, 6-CH_3_ (as in **9**), 7-COOCH_3_, and 7-CH_3_ on the imidazo­[1,2-*a*]­pyridine scaffold enhanced the antiproliferative potency against MV4–11, supporting **9** as the most promising candidate. Another relevant observation is that simultaneous substitution at positions 6 and 7 of the imidazo­[1,2-*a*]­pyridine ring with bromo and methyl groups slightly reduced both FLT3-ITD inhibition and antiproliferative activity against MV4–11 cells. Molecular docking studies showed that **9** occupies the same ATP-binding site in the DFG-out conformation as quizartinib, forming two hydrogen bonds between the urea NH group and Glu661, while the imidazo­[1,2-*a*]­pyridine moiety engages in a hydrogen bond with Cys697. Compound **9** also exhibited antiproliferative activity against MOLM-13 cells (IC_50_ = 18.5 nM), comparable to that of quizartinib (IC_50_ = 10.0 nM). In other cancer cell lines, both compounds showed IC_50_ values of >1000 nM. No significant toxicity was observed in the heart, liver, spleen, lung, kidney, or brain tissues at 100 and 300 mg·kg^–1^, suggesting a favorable safety profile. However, despite its *in vivo* safety, **9** did not achieve significant tumor growth inhibition in the MV4–11 xenograft model, likely due to its suboptimal pharmacokinetic properties. Overall, although **9** lacked *in vivo* efficacy, its potent *in vitro* activity, high selectivity, and encouraging safety profile support its identification as a promising lead compound for the development of new FLT3 inhibitors targeting acute myeloid leukemia.^71^

**Figure 9:**
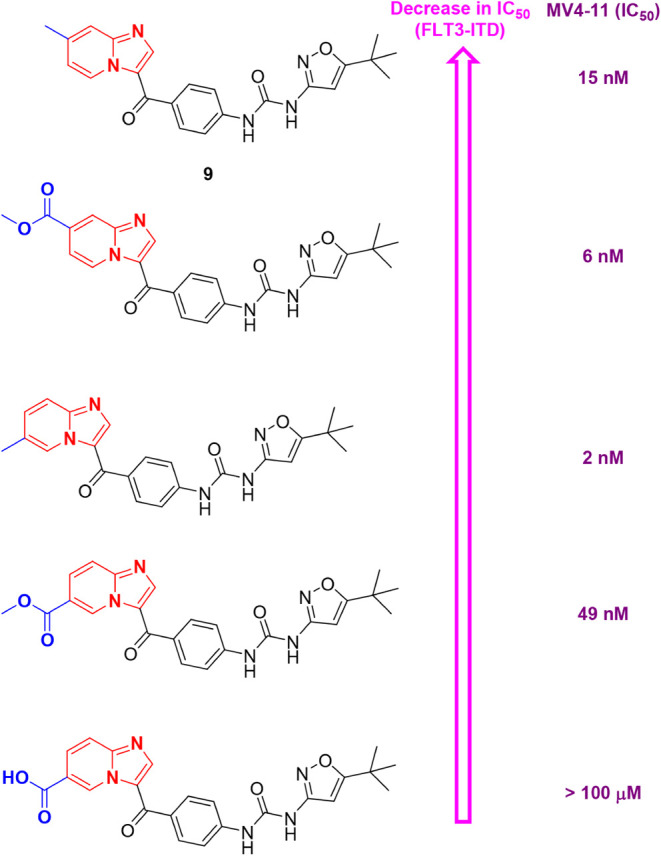
Structural comparison between **9** and its analogs versus their antiproliferative activity against MV4–11 cells.

Sarkar and coworkers synthesized a series of *N*-linked imidazo­[1,2-*a*]­pyridine-benzoheterobicyclic molecular hybrids with potential antiproliferative properties. The lead compound, **10**, inhibited the proliferation of cervical and breast cancer cells, exhibiting IC_50_ values of 7.4 and 9.3 μM against HeLa (cervical adenocarcinoma) and MCF-7 cells, respectively (Figure 10). Compound **10** suppressed DMSO-induced tubulin polymerization and promoted the depolymerization of cellular microtubules. A significant increase in the G2/M cell population was observed following treatment with 15 and 30 μM, with 35 and 33% of cells arrested in this phase, respectively. This finding indicates that the compound impedes cell cycle progression by inducing mitotic arrest. Consequently, a marked reduction in the G_0_/G_1_ population was detected, 52 and 55% at 15 and 30 μM, respectively, compared to the positive control (vinblastine, 20 nM), which displayed 70%. Molecular docking studies revealed that **10** exhibits binding affinity for the colchicine site of tubulin, interacting with key active-site residues, including Ser C:178, Thr C:179, Ala C:180, Val D:238, Cys D:241, Leu D:242, Leu D:248, Ala D:250, Lys D:254, Leu D:255, Asn D:258, and Met D:259. The docking score for **10** was −8.789 kcal·mol^–1^, compared to −9.372 kcal·mol^–1^ for colchicine. Another relevant structure–activity observation is that a chlorine substituent at the C-6 position of the imidazo­[1,2-*a*]­pyridine ring enhances the biological activity of the lead compound, whereas a bromine atom at the same position diminishes it. Moreover, the presence of electron-donating groups at C-6 generally correlated with reduced activity.^72^

**Figure 10:**
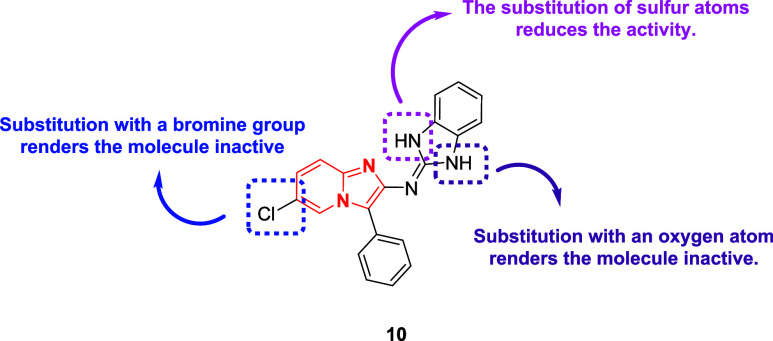
Structure of compound **10** with antiproliferative activity, focusing on observations based on the SAR.

Mahmoud and coworkers developed new imidazo­[1,2-*a*]­pyridine-triazole hybrid compounds as anticancer agents targeting MMP13 (matrix metalloproteinase-13). The lead compound demonstrated superior antiproliferative activity compared to doxorubicin, the positive control. Compound **11** exhibited IC_50_ values of 5.86 and 23.07 μM against PC-3 (prostate cancer) and MDA-MB-231 cells, respectively, whereas doxorubicin showed IC_50_ values of 9.37 and 29.24 μM for the same cell lines (Figure 11). Both compounds were further evaluated using WI-38 (human diploid lung fibroblasts), where the lead compound displayed no detectable cytotoxicity (IC_50_ > 50 μM), whereas doxorubicin exhibited higher cytotoxicity than in the cancer cell lines tested (IC_50_ = 6.51 μM). It is important to note that the second most active compound exhibited antiproliferative activity that was 2.6 and 1.3 times lower, respectively, in the PC-3 and MDA-MB-231 cell lines, whereas this compound did not contain the chlorinated substituents found in the original compound. Target prediction analysis identified MMP13 as the primary target. Molecular docking studies revealed that **11** showed a binding energy of −8.13 kcal·mol^–1^, engaging in π-alkyl, π-π, metal-acceptor, and cation-π interactions, whereas the cocrystallized ligand exhibited a binding energy of −9.99 kcal·mol^–1^ and formed π-sigma, π-alkyl, π–π, hydrogen-bond, and metal-acceptor interactions. The mechanism of action of **11** was experimentally validated through wound-healing inhibition assays and by demonstrating the suppression of MMP13 expression in PC-3 cell culture supernatants.^73^

**Figure 11:**
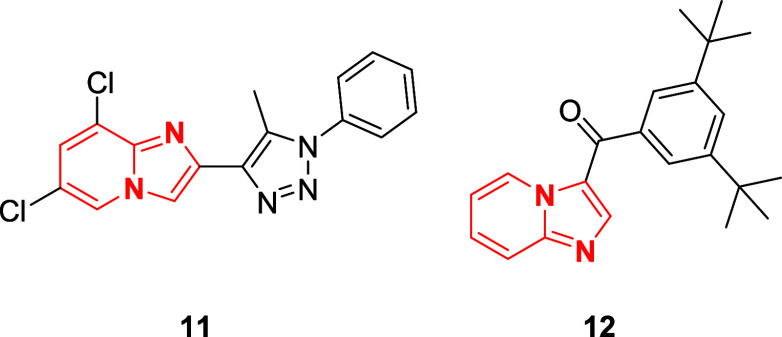
Structures of the compounds **11** and **12** with antiproliferative activity.

Luo and colleagues synthesized imidazo­[1,2-*a*]­pyridines through a (3 + 2) cycloaddition using NaIO_4_/TBHP (*tert*-butyl hydroperoxide) and evaluated them as anticancer agents. The compounds were tested against MOLM-13 and MV4–11 cell lines. The lead compound, **12**, exhibited IC_50_ values of 8.53 and 9.73 μM against MOLM-13 and MV4–11 cells, respectively, whereas the positive control, cabozantinib, showed IC_50_ values of 0.045 and 0.003 μM, respectively (Figure 11). The SAR indicated that the substituents 3,5-di-*tert*-butylphenyl >3,4-dioxanephenyl >3,4,5-trimethoxyphenyl yielded the highest antiproliferative activity, with IC_50_ values below 20 μM. The remaining compounds in this series exhibited IC_50_ values above 32 μM against MV4–11. In the MOLM-13 cell line, two additional compounds exhibited moderate activity, with IC_50_ values between 24 and 30 μM, bearing 4-chlorophenyl > phenyl groups. Molecular docking studies targeting FLT3-ITD, comparing **12** with quizartinib, revealed *E*_*score*_ values (kcal·mol^–1^) of −10.86 and −6.32, respectively. **12** improved the occupation of the ATP adenosine-binding site, forming a key hydrogen bond with the Cys694 residue through the acceptor nitrogen atom of the imidazo­[1,2-*a*]­pyridine scaffold. These findings suggest that further optimization could enhance the development of novel FLT3 inhibitors.^21^

An important study investigated isoquinoline-imidazo­[1,2-*a*]­pyridine-linked sulfonyl derivatives as potential anticancer agents. Gunuguntla and colleagues evaluated these compounds *in vitro* for their inhibitory effects on EGFR kinase and their antiproliferative activities against two breast cancer cell lines, MCF-7 and MDA-MB-231 (Table 1). Four compounds (**13**-**16**) showed antiproliferative activity below 10 μM. In addition, the compounds were tested against the nontumorigenic MCF-10A cell line, and the results were comparable to those of the positive control, erlotinib, with **14** and **16** showing the least cytotoxicity. All four lead compounds contained either (trifluoromethyl)­phenyl or (trifluoromethoxy)­phenyl groups, differing only in their substitution at positions 3 or 4. The activity against MCF-7 cells was higher than that against MDA-MB-231 cells. The SAR analysis revealed that the imidazo­[1,2-*a*]­pyridine scaffold is primarily responsible for the anticancer activity, the isoquinoline moiety confers drug-like properties, the sulfonyl–pyridine group enhances the activity, and the aromatic units modulate the lipophilicity. Furthermore, electron-withdrawing groups increased the anticancer activity, with −CF_3_ and −OCF_3_ outperforming −F and −Cl. EGFR inhibition assays showed that all four lead compounds exhibited values close to that of erlotinib (430 nM), with **15** (240 nM) and **14** (330 nM) being the most potent. Overall, **14** exhibit the most favorable performance. Molecular docking studies provided additional insights, including the binding energies (kcal·mol^–1^) for the interaction with EGFR. Compounds **14** and **15** were the most potent, with values of −10.63 and −10.97, respectively. Compound **14** formed three hydrogen bonds with residues Lys721, Asp831, and Phe832, whereas **15** formed four hydrogen bonds with Lys721, Asp831, Phe832, and Arg817. The positive control, erlotinib, showed a binding energy of −8.25 and formed two hydrogen bonds with Met769 and Cys773, respectively. Taken together, these findings highlight **14** as a particularly promising compound and support its potential as a lead candidate for the development of novel anticancer agents.^74^

**Table 1:**
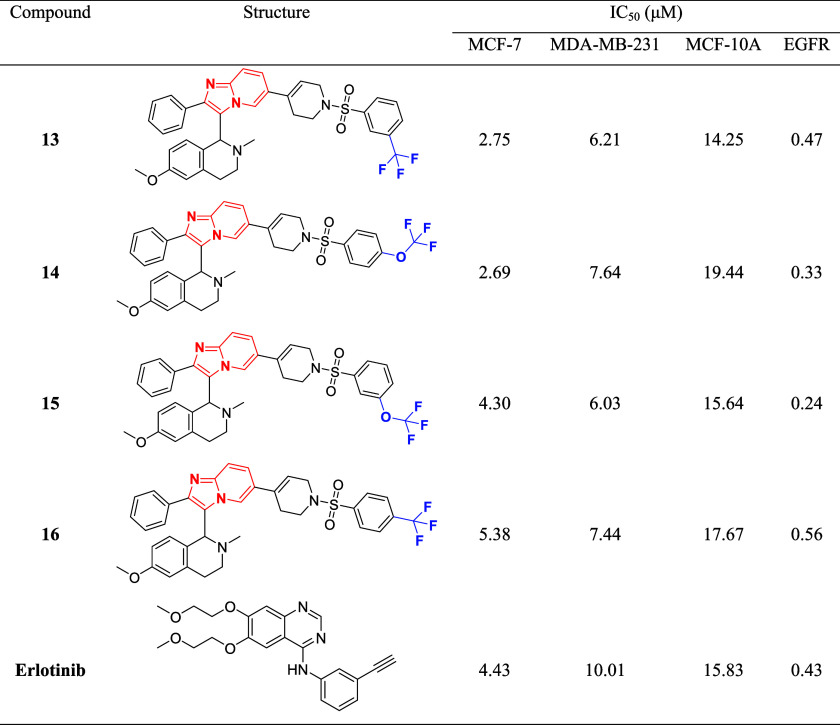
Comparison of the Isoquinoline–Imidazo­[1,2-*a*]­pyridine-Linked Sulfonyl Derivatives with the Highest Antiproliferative Activity against the MCF-7 and MDA-MB-231 Cancer Cell Lines, Their Effects on the Nontumorigenic MCF-10A Breast Cell Line, and Assessment of Their EGFR Inhibitory Activity

Firouzi and colleagues synthesized and evaluated the cytotoxic activities of imidazo­[1,2-*a*]­pyridine carbohydrazide derivatives. The most active compound against MCF-7 and HT-29 cell lines exhibited IC_50_ values of 22.6 and 13.4 μM, respectively (Figure 12). Compound **17** (4-bromophenyl) showed no activity against the K562 chronic myeloid leukemia cell line or the nontumorigenic Vero epithelial kidney cells derived from the African green monkey. The positive controls, cisplatin and doxorubicin, displayed IC_50_ values of 25.9, 22.4, 9.4, and 10.6 μM, and 0.3297, 0.7503, 0.0449 nM, and 2.6 μM, respectively, against MCF-7, HT-29, K562, and Vero cells. Considering an activity threshold of IC_50_ < 50 μM, only a subset of the derivatives showed relevant antiproliferative effects. Derivatives bearing phenyl, 4-fluorophenyl, 4-chlorophenyl, and 4-methyl substituents exhibited no antiproliferative activity. Cell cycle analysis revealed that compound **17** increased the proportion of MCF-7 cells in the G_0_/G_1_ phase and induced apoptosis in this cell line. Molecular docking studies, based on the prediction of platelet-derived growth factor receptor alpha (PDGFRA) as a potential target, showed that the amide group of **17** formed two hydrogen bonds with residues Glu644 and Asp836 and established 11 hydrophobic contacts with the receptor, which may enhance ligand–protein binding affinity. Overall, this study provides a promising starting point for future investigations of hydrazinyl imidazo­[1,2-*a*]­pyridine derivatives.^75^

**Figure 12:**
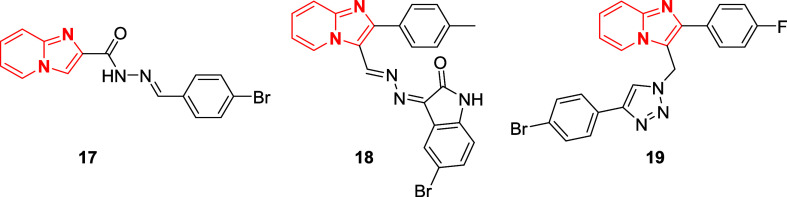
Structures of compounds **17**-**19** that exhibited anticancer activity.

Elkotamy and coworkers developed imidazo­[1,2-*a*]­pyridine derivatives linked to an indolinone scaffold as potential VEGFR-2 (vascular endothelial growth factor receptor 2) inhibitors. The most active compound, **18**, exhibited an IC_50_ value of 0.33 μM against VEGFR-2, comparable to that of the positive control sunitinib (IC_50_ = 0.17 μM) (Figure 12). In addiction to enzymatic assays, the compounds were evaluated for their antiproliferative activity against MCF-7 and MDA-MB-231 breast cancer cell lines. Compound **18** exhibit an IC_50_ of 10.88 μM, comparable to that of cisplatin (IC_50_ = 11.50 μM). Notably, the nontumorigenic breast epithelial cell line MCF-10A, **18** showed an IC_50_ > 50 μM, indicating a lack of cytotoxicity; in contrast, cisplatin exhibited an IC_50_ of 21.75 μM, highlighting its lower cellular selectivity. Cell cycle analysis in MDA-MB-231 cells revealed that **18** induced arrest at the G_0_/G_1_ phase, increasing the cell population to 66.18% compared to 59.48% in the control. Additionally, **18** markedly triggered apoptosis, the and levels of cytochrome c, caspase-8, and caspase-9 increased by 12.23-, 8.84-, and 4.71-fold, respectively. The compound also upregulated BAX and downregulated Bcl-2, consistent with the activation of both intrinsic and extrinsic apoptotic pathways. Taken together, these findings highlight **18** as a promising and selective anticancer candidate, combining effective VEGFR-2 inhibition, significant antiproliferative activity in tumor cell lines, and minimal cytotoxicity toward noncancerous cells. Therefore, *in vivo* studies are warranted to validate their antitumor potential and guide future structural optimizations.^76^

Albayrak Halac and coworkers synthesized a new series of imidazo­[1,2-*a*]­pyridine-triazole hybrids as potential antitumor agents. The compounds were evaluated *in vitro* against the cancer cell lines MCF-7, A549 (nonsmall cell lung cancer), and HepG2 (human hepatocellular carcinoma). The 4-bromophenyl derivative **19** was identified as the most potent analog based on its antiproliferative activity, with IC_50_ values of 25.2, 19.5, and 36.9 μM against MCF-7, A549, and HepG2 cells, respectively (Figure 12). A potentially concerning observation is that **19** displayed relatively low selectivity, showing cytotoxicity toward normal human fibroblasts (MRC-5, IC_50_ = 24.0 μM). Tamoxifen, the positive control, demonstrated stronger activity, with IC_50_ values of 8.6, 7.16, and 8.8 μM in the corresponding cancer cell lines, thus remaining the most active compound in the study. Notably, two additional derivatives exhibited superior selectivity profiles: the phenyl analog showed the best activity against A549 (IC_50_ = 16.5 μM), and the 4-methylphenyl analog displayed improved potency against MCF-7 (IC_50_ = 20.6 μM). Importantly, neither compound exhibited cytotoxicity toward the normal MRC-5 cell line. Compound **19** and the remaining derivatives were subjected to molecular docking studies against several biological targets, with CDK2 (cyclin-dependent kinase 2) emerging as the most relevant. Compound **19** exhibited a calculated binding affinity of −10.9 kcal·mol^–1^, which was superior to that of the reference drug sorafenib (−9.2 kcal·mol^–1^). The compound formed hydrogen bonds with residues Thr14, Lys129, Gln131, and Asn132 within the CDK2 active site. However, *in silico* data also suggested possible solubility issues for compound **19**. Collectively, these results indicate that **19** is a promising, albeit not yet optimized, CDK2-targeting scaffold. Further studies are required to validate its *in vitro* activity against the proposed molecular targets and to conduct *in vivo* investigations to assess its therapeutic potential.^77^

A particularly interesting study focused on the design, synthesis, and anticancer evaluation of isoxazole-imidazo­[1,2-*a*]­pyridine amide derivatives. Jami and coworkers synthesized ten derivatives and investigated their antiproliferative effects against a panel of four human cancer cell lines (PC-3 and DU-145 (prostate cancer), A549 and MCF-7) using etoposide as a reference drug. Among the synthesized compounds, **20**-**22** exhibited markedly superior antiproliferative activity across all the tested cancer cell lines compared to etoposide (Table 2). Notably, **20** exhibit the lowest IC_50_ values across all four cell lines, emerging as the most potent analog. From a SAR perspective, an interesting trend was observed within this subset of compounds: moving from **20** to **22**, one methoxy group was removed sequentially at each step. The most active compound contained a 3,4,5-trimethoxy substitution pattern, whereas the least active compound, although still significantly potent, was the 4-methoxy analog. This trend highlights the importance of progressive methoxy substitution in modulating the antiproliferative activity. Considering the remaining derivatives in the series, the following increasing order of antiproliferative potency was established: 3,5-dinitro <4-bromo <4-cyano <4-chloro <4-nitro <4-methyl <4-(dimethyl)­amino. These findings support further investigation into the physicochemical properties, pharmacokinetic behavior, and *in vivo* performance of this chemical class, given the strong *in vitro* anticancer potential of these isoxazole-imidazo­[1,2-*a*]­pyridine amide derivatives.^78^

**Table 2:**
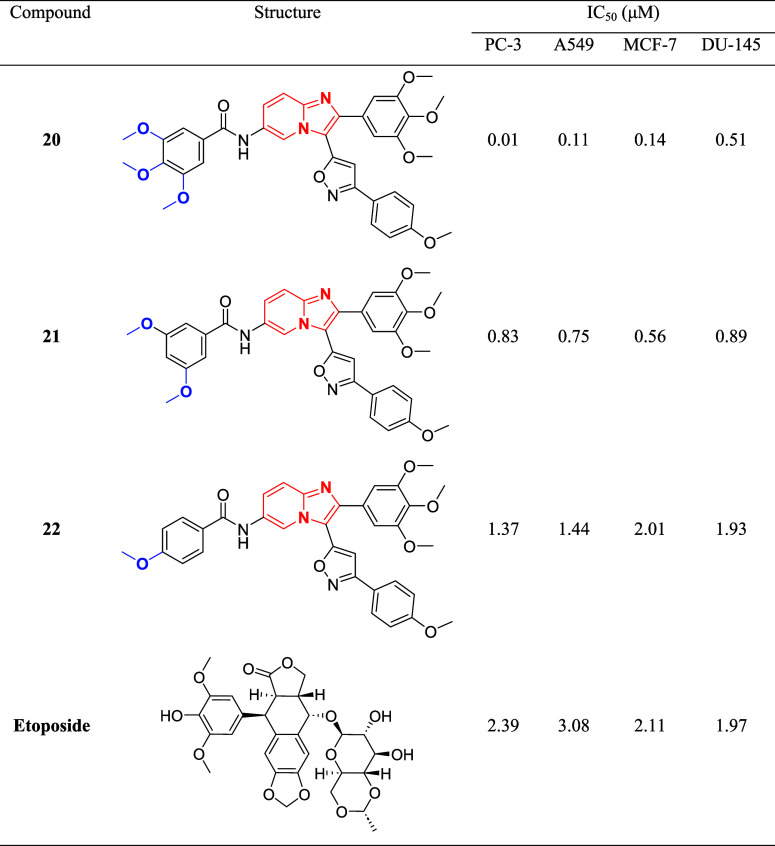
Structures of the Isoxazole-Imidazo­[1,2*a*]­pyridine Derivatives Exhibiting the Most Potent Antiproliferative Activity across All Four Tested Cancer Cell Lines

Kaya and colleagues synthesized a new series of imidazo­[1,2-*a*]­pyridine-based heterocycles bearing *S*-alkyl/aryl substituents. Fourteen novel derivatives were obtained and evaluated for cytotoxicity against the tumor cell lines A549, MCF-7, and HepG2, as well as the healthy NIH/3T3 mouse embryonic fibroblast line. The lead compound, **23**, exhibit the most potent antiproliferative activity across tested tumor cell lines. The IC_50_ values were 3.16, 7.00, and 6.00 μM against HepG2, A549, and MCF-7 cells, respectively, and 10 μM in the nontumorigenic NIH/3T3 line, indicating mild cytotoxicity (Figure 13). In contrast, the positive control exhibited IC_50_ values >100 μM against HepG2, MCF-7, and NIH/3T3 cells, showing activity only toward A549 cells (IC_50_ = 40 μM). The authors further assessed the ability of **23** to inhibit DNA synthesis and cell proliferation in HepG2 cells, *in vitro*. Compound **23** induced 9.50, 27.75, and 57.86% inhibition at IC_50_/2, IC_50_, and 2 × IC_50_, respectively, whereas cisplatin induced 30.49, 43.91, and 48.78% inhibition under the same conditions. Molecular docking studies revealed binding energies of −7.8 and −10.7 kcal·mol^–1^ for **23** with caspase-3 and caspase-9, respectively. Collectively, these findings highlight the promising anticancer potential of **23** and warrant further investigation to confirm its efficacy and support future *in vivo*.^79^

**Figure 13:**
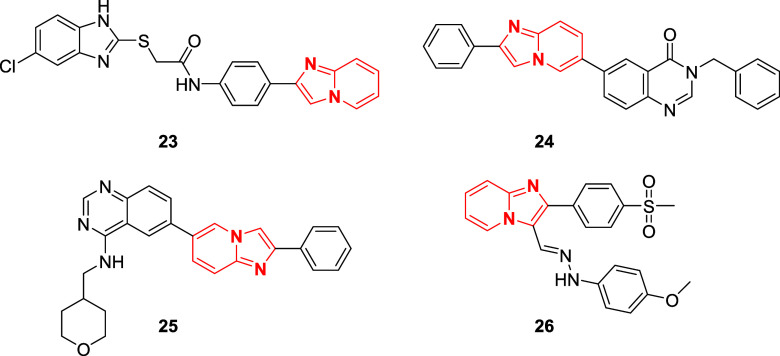
Structures of compounds **23**-**26** with antiproliferative activity.

Fan and collaborators developed a series of 6-(imidazo­[1,2-*a*]­pyridin-6-yl)­quinazolin-4­(3*H*)-one derivatives as potent anticancer agents with dual inhibitory activity against Aurora kinases and receptor tyrosine kinase-like orphan receptor 1 (ROR1). The lead compound, **24**, displayed IC_50_ values of 90, 70, 80, and 50 nM against the cancer cell lines HCC827 (nonsmall cell lung cancer), MHCC-97H (hepatocellular carcinoma), BGC823 (gastric cancer), and SH-SY5Y (neuroblastoma), respectively, and an IC_50_ value of 490 nM against the normal human liver cell line LX2 (Figure 13). As a reference, the Aurora A inhibitor ENMD-2076 exhibited IC_50_ values of 350, 360, 310, and 630 nM against the same cancer cell lines, and its activity was not determined for LX2. **24** also inhibited Aurora A and Aurora B with IC_50_ values of 84.41 nM and 14.09 nM, respectively. Moreover, **24** induced G_2_/M cell cycle arrest at 24 h and promoted polyploidy after 48 h. This compound further inhibited ROR1 expression and triggered apoptosis. In SH-SY5Y cells, **24** induced mitotic catastrophe and apoptosis, as evidenced by increased levels of pro-apoptotic proteins, including cleaved PARP, cleaved caspase-9, and p53, as shown by Western blot analysis. In an SH-SY5Y xenograft model, **24** suppressed tumor growth by 46.31% at 10 mg·kg^–1^ and 52.66% at 20 mg·kg^–1^. Collectively, these findings identify **24** as a novel dual inhibitor of ROR1 and Aurora kinases, exhibiting potent antitumor activity both *in vitro* and *in vivo*. This compound represents a promising therapeutic candidate for cancers characterized by aberrant ROR1 and Aurora kinase expression through the simultaneous blockade of both targets.^80^

Li and coworkers evaluated a series of 6-(imidazo­[1,2-*a*]­pyridin-6-yl)­quinazoline derivatives as potential anticancer agents targeting PI3Kα (phosphatidylinositol 3-kinase alpha isoform). In an *in vitro* antitumor study, most compounds displayed antiproliferative activity, with lead compound **25** emerging as the most potent. The IC_50_ values ranged from 0.09 to 0.43 μM against HCC827, A549, SH-SY5Y, HEL (acute erythroid leukemia), and MCF-7 cell lines (Figure 13). The positive control, HS-173, a selective PI3Kα inhibitor, exhibit IC_50_ values ranging from 3.90 to 10.71 μM. Furthermore, **25** induced G2/M cell-cycle arrest and apoptosis in HCC827 cells through PI3Kα inhibition, with an IC_50_ value of 1.94 nM, whereas the positive control HS-173 exhibited an IC_50_ value of 3.72 nM. Molecular docking studies revealed that the compound interacts with PI3Kα via two hydrogen bonds with residues Lys802 and Gln859, in addition to hydrophobic, π–π, and π–sulfur interactions. This binding mode is consistent with that of other PI3Kα inhibitors. Taken together, these findings suggest that **25** may serve as a promising lead compound for the development of new PI3Kα inhibitors.^81^

Ismael and colleagues reported novel imidazo­[1,2-*a*]­pyridine derivatives as potential anticancer agents. The cytotoxic activities of these compounds were evaluated against several human cancer cell lines. Four derivatives with the most promising cytotoxic profiles were selected, and their IC_50_ values were determined against K562 cells and the normal WI38 human lung fibroblast line. Compound **26**, which contains a hydrazone scaffold and a 4-OCH_3_ substituent, exhibited IC_50_ values of 1.1 and 11.5 μM, respectively, whereas the positive control gefitinib exhibited IC_50_ values of 6.1 and 17.1 μM (Figure 13). Based on these values, **26** was more cytotoxic to K562 cells but also displayed increased cytotoxicity toward normal cells. A structural analog of **26**, featuring a hydrazone core and a 4-CH_3_ substituent, exhibit IC_50_ values of 2.9 and 24.2 μM against K562 and WI38 cells, respectively, demonstrating notable antiproliferative potency while being less cytotoxic than **26**. Both compounds were assessed for EGFR inhibitory activity, yielding IC_50_ values of 70 and 120 nM for **26** and the 4-CH_3_ analog, respectively, compared to 50 nM for the positive control. Additionally, their anti-inflammatory activity was evaluated: **26** displayed IC_50_ values of 15.0 and 1.1 μM against COX-1 (cyclooxygenase-1) and COX-2 (cyclooxygenase-2), respectively, with a selectivity index (SI) of 13.8. The reference drugs indomethacin and celecoxib showed IC_50_ values of 1.6 and 4.9 μM (SI = 0.3) and 15.0 and 0.9 μM (SI = 17.6), respectively, indicating that **26** also exhibits remarkable COX-2-selective anti-inflammatory activity. The apoptosis-inducing potential of the most active compound, **26**, was demonstrated to trigger pre-G_1_ apoptosis and induce G_1_ cell cycle arrest in K562 leukemia cells. Moreover, **26** markedly upregulated caspase-3 expression (6.98-fold) compared with than in the control. Physicochemical parameters were calculated, confirming the compound’s favorable oral bioavailability.^82^

An interesting study explored the use of 2,6,8-substituted imidazo­[1,2-*a*]­pyridine derivatives as potential PI3Kα inhibitors. Chen and coworkers designed these compounds to evaluate their activity against PI3Kα and across a panel of PI3Kα-dependent cancer cell lines, including SKOV-3, T47D (breast cancer), NCI-H1975 (nonsmall-cell lung cancer), NCI-H460 (nonsmall-cell lung cancer), and MCF-7. The lead compound, **27**, exhibit IC_50_ values of 26.6, 7.9, 32.1, 17.7, and 9.4 μM, whereas the positive control, alpelisib, exhibited IC_50_ values of 9.7, 0.46, 2.7, 8.9, and 2.2 μM (Figure 14). Compound **27** exhibited promising antiproliferative activity against the breast cancer cell lines (IC_50_ < 10 μM). Compound **27** was further evaluated for PI3Kα inhibitory activity, yielding an IC_50_ value of 150 nM, compared to 7.4 nM for alpelisib. In T47D cells, N**27** induced S-phase cell cycle arrest. To probe its *in vitro* ADME properties, **27** showed acceptable stability in human and mouse liver microsomes. Membrane permeability was assessed using a parallel artificial membrane permeability assay (PAMPA), which revealed that the compound was highly permeable. To minimize potential drug–drug interactions, **27** was examined for inhibitory activity against the major cytochrome P450 isoforms (CYP1A2, CYP2D6, CYP2C9, CYP2C19, and CYP3A4), showing minimal or negligible inhibition across all enzymes. Molecular docking studies indicated that **27** forms two hydrogen-bond interactions with Lys802 and establishes hydrophobic contacts with Ile848, Val851, Thr856, and Asp933. Overall, additional optimization efforts are required to develop more potent PI3Kα inhibitors based on this scaffold.^83^

**Figure 14:**
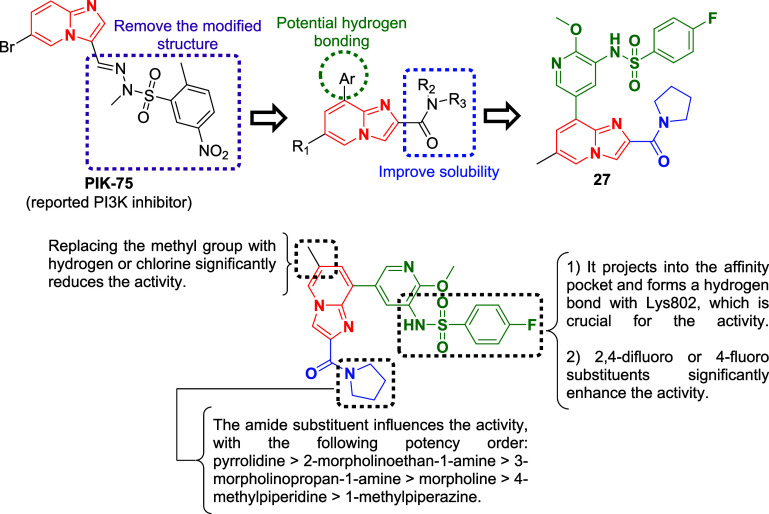
Structural optimization of the PI3K inhibitor PIK-75 lead to the development of a potent and selective PI3Kα inhibitor, highlighting the relationship between the key structural features of the **27** scaffold and its anticancer activity.

Dos Santos and colleagues evaluated selenylated imidazo­[1,2-*a*]­pyridines in the HepG2 cancer cell line and *in vivo* models. The lead compound, **28** (3-((2-methoxyphenyl)­selanyl)-7-methyl-2-phenylimidazo­[1,2-*a*]­pyridine), demonstrated remarkable cytotoxic activity against HepG2 cells, with an IC_50_ of 0.03 μM (Table 3). Compared with the normal fibroblast cell line McCoy (IC_50_ = 12.0 μM), **28** displayed an exceptionally high selectivity index (400), far surpassing that of the other derivatives, **29** and **30**, as well as the positive control doxorubicin. Compound **29** exhibited 28-fold lower antiproliferative activity than compound **28**, whereas compound **30** was 733-fold less potent. At noncytotoxic concentrations, **28** decreased Bcl-xL protein levels and increased p53 levels, leading to the inhibition of cell proliferation and induction of apoptosis. Moreover, the compound reduced extracellular signal-regulated kinase (ERK1/2) protein levels and modulated key regulators of the cell cycle, tumor growth, and survival, including cyclin B1 and CDK2, resulting in a reduction in the number of cells in the S phase. Compound **28** also enhanced reactive oxygen species (ROS) generation, impaired antioxidant defense, and triggered DNA fragmentation. *In vivo* assays, **28** significantly inhibited the growth of Ehrlich ascites tumors in mice, achieving a 46% inhibition rate at 1 mg·kg^–1^, in addition to increasing survival time and suppressing angiogenesis. Collectively, these findings identify **28** as a promising candidate for the development of novel therapeutic agents targeting hepatocellular carcinoma.^84^

**Table 3:**
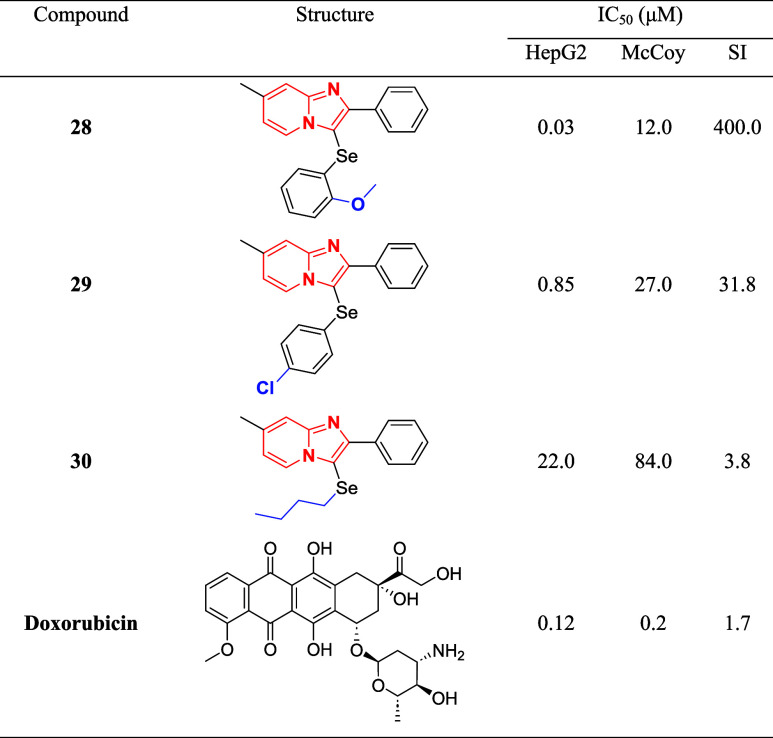
Structures of the Three Most Active Compounds Identified in this Study, Particularly **28**, Which Exhibited Exceptionally Potent Antiproliferative Activity and a High Selectivity Index Relative to the Normal Cell Line

Singh and collaborators synthesized a series of 3-N/O/S-methyl-imidazo­[1,2-*a*]­pyridine derivatives to evaluate their anticancer activity against the cancer cell lines MCF-7, MDA-MB-231, MiaPaca-2 (pancreatic cancer), A549, PC-3, and HCT-116 (colorectal cancer), as well as the normal cell line HEK-293. The lead compound of the study, **31**, exhibited IC_50_ values of 3.5, 8.7, and 8.9 μM against HCT-116, A549, and MiaPaca-2 cancer cells, respectively, and showed time-dependent inhibition in HCT-116 cells while displaying no toxicity toward normal cells (Figure 15). SAR analysis revealed that the presence of a methyl substituent at position 6 of the imidazo­[1,2-*a*]­pyridine ring significantly enhanced the antiproliferative activity, whereas the presence of hydrogen, chlorine, or bromine at the same position reduced potency. Furthermore, replacing sulfur with oxygen or nitrogen abolished the activity in HCT-116 cells. **31** induced apoptosis in the HCT-116 cell line, as evidenced by ROS generation, loss of mitochondrial membrane potential (MMP), and characteristic morphological changes in the cells. Its anticancer potential is associated with the upregulation of the PTEN gene, downregulation of the AKT pathway, and subsequent caspase-3 cleavage. Molecular docking studies demonstrated that **31** exhibited strong binding to the PI3Kα active site, with a docking score of −6.932 kcal·mol^–1^, forming a key hydrogen bond with Val851. In addition, superposition with the cocrystallized ligand alpelisib revealed a similar binding orientation in the active site.^85^

**Figure 15:**
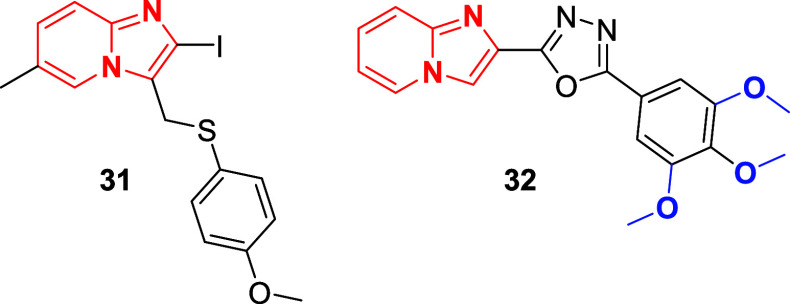
Structures of compounds **31** and **32** that exhibited anticancer activity.

Sigalapalli and coworkers synthesized a series of imidazo­[1,2-*a*]­pyridine-oxadiazole hybrids as potential antiproliferative agents. The lead compound, **32**, demonstrated pronounced cytotoxicity and excellent cytoselectivity toward cancer cell lines compared to normal cells. **32** exhibited IC_50_ values of 2.8, 3.5, and 4.5 μM against A549, PC-3, and DU-145 cells, respectively, while showing no cytotoxicity toward the normal HEK-293 cell line (IC_50_ > 80 μM) (Figure 15). SAR analysis indicated that electron-donating groups on the phenyl ring attached to the 1,3,4-oxadiazole scaffold enhanced the bioactivity of the compounds relative to their unsubstituted analogs. **32** induced apoptosis in A549 cells, as demonstrated by Annexin V/propidium iodide double staining, with 4.3% viable cells and 86.5% apoptotic cells (including early and late apoptosis). In addition, **32** caused cell cycle arrest at the G_0_/G_1_ and G_2_/M phases in A549 cells. Compound **32** significantly inhibited tubulin polymerization, with an IC_50_ of 3.45 μM, and effectively bound to calf thymus DNA (CT-DNA). Molecular docking studies showed that **32** binds at the α/β-tubulin interface, where the oxadiazole nitrogen acts as a hydrogen-bond acceptor, forming two interactions with Asn249 and Ala250 at distances of 3.54 and 3.17 Å, respectively. The 3,4,5-trimethoxyphenyl ring establishes a cation−π interaction with Lys254 in the active site. Finally, the physicochemical and ADMET properties of compound **32** fell within the acceptable ranges for drug-like molecules.^86^

### 3.2 Antifungal

Puumala and colleagues optimized small-molecule inhibitors targeting Yck2 (yeast casein kinase) as a potential strategy to combat *Candida albicans* infections. Their study reported the optimization of GW461484A, a known Yck2 inhibitor, through two complementary approaches: the synthesis of bioisosteres featuring the imidazo­[1,2-*a*]­pyridine scaffold and modification of the “R” group on the pyrazolo­[1,5-*a*]­pyridine core of GW461484A (Figure 16). Two 6-cyano-substituted derivatives were identified, exhibiting improved pharmacological properties while maintaining whole-cell bioactivity and selectivity toward fungal Yck2 over human CK1α. Of the 24 synthesized compounds, 20 inhibited purified *C. albicans* Yck2 with IC_50_ values below 1.0 μM. Four derivatives belonging to the pyrazolo­[1,5-*a*]­pyridine series, bearing 6-CF_3_, 5-CF_3_, 4-CH_3_, or 4-CN substituents, displayed IC_50_ values above the highest tested concentration, indicating that these modifications were poorly tolerated. In general, the imidazo­[1,2-*a*]­pyridine bioisosteres were less selective for *C. albicans* Yck2 than their pyrazolo­[1,5-*a*]­pyridine counterparts, with a mean fungal selectivity of approximately 7-fold compared to ∼23-fold for the latter series. Interestingly, for both the 6-substituted imidazopyridine and pyrazolopyridine series, several analogs exhibited robust activity against *C. albicans*, particularly those bearing 6-cyano (**33** and **35**) or 6-fluoro (**34** and **36**) substituents. As expected, the parent compound GW461484A and other methyl-substituted analogs were highly unstable. Among the metabolically stable derivatives, two 6-substituted pyrazolo­[1,5-*a*]­pyridines (6-CN and 6-F) and two 6-substituted imidazo­[1,2-*a*]­pyridine bioisosteres (6-CN and 6-F) exhibited promising target potency and whole-cell antifungal activity, justifying their prioritization for further characterization. Since the present study focuses on imidazo­[1,2-*a*]­pyridine derivatives, attention was directed toward compounds **33** and **34**. Although these bioisosteres were less selective for fungal Yck2 than the pyrazolopyridine analogs, they demonstrated fungal selectivity comparable to that of GW461484A, with 8- and 12-fold differences, respectively. Structural studies revealed that these compounds occupied positions nearly identical to those of GW461484A within the Yck2 ATP-binding site (PDB: 6U6A). The 6-cyano substituents extended toward Asp167, a residue highly conserved among CK1 orthologs, forming a favorable N–O polar interaction, which is interaction not achievable with the 6-methyl group of GW461484A. The Yck2 P-loop in complexes **33** and **34** adopted conformations closely resembling that of the GW461484A-bound structure, with Glu52 positioned 4–5 Å from the 6-substituent and Phe55 located >10 Å away from the ligands. *In vivo* efficacy studies showed that both 6-cyano analogs exhibited standalone activity against *C. albicans* strains resistant to first-line echinocandin antifungals and enhanced the noncurative echinocandin treatment response. Collectively, these findings validate Yck2, a member of the casein kinase 1 (CK1) family, as a promising antifungal target and support the further development of inhibitors acting through this previously unexplored mechanism of action.^87^

**Figure 16:**
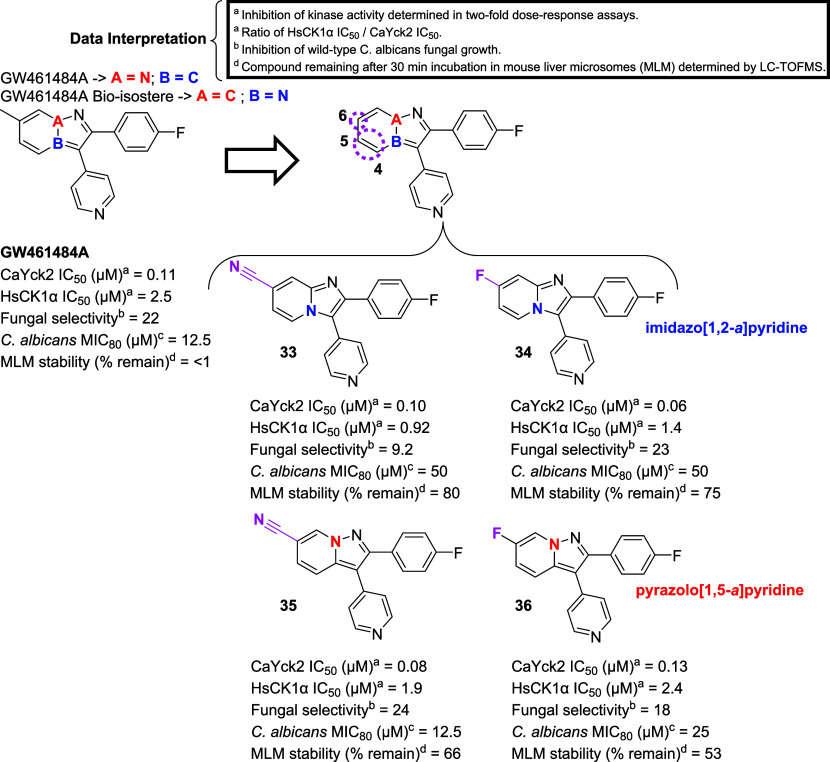
Structural and antifungal activity comparisons of the most active compounds, varying the core scaffold from imidazo­[1,2-*a*]­pyridine to pyrazolo­[1,5-*a*]­pyridine.

An interesting study investigated the antifungal and antinematicidal activities of a newly designed series of compounds. Lu and collaborators synthesized and evaluated thirty-two structurally planned derivatives. Among them, compound **37** exhibited an EC_50_ (half-maximal effective concentration) of 3.33 mg·L^–1^ against *Rhizoctonia solani*, outperforming the positive control fluopyram, which showed an EC_50_ of 112.74 mg·L^–1^ *in vitro* (Figure 17). In pot assays, *in vivo* evaluation of **37** demonstrated protective efficacies of 83.95% and 91.26% at 100 and 200 mg·L^–1^, respectively, values comparable to carbendazim (86.90% and 96.67%, respectively). The curative efficacy of **37** was 65.86% and 78.69% at the same concentrations, slightly lower than that of carbendazim (76.86% and 89.30%). At higher doses, **37** effectively suppressed the appearance of characteristic grayish-brown necrotic sheath lesions. The study also assessed antinematicidal activity and identified **38** as the lead compound. Compound **38** exhibited an *in vitro* LC_50_ (median lethal concentration) value of 40.31 mg·L^–1^ against *Caenorhabditis elegans* (48 h), which was superior to that of fosthiazate (73.26 mg·L^–1^) but not as potent as that of fluopyram (7.65 mg·L^–1^), both of which were used as positive controls. *In vivo* assays against *Meloidogyne incognita* showed that **38** achieved efficacies of 67.44%, 75.58%, and 83.72% at 50, 100, and 200 mg·L^–1^, respectively, outperforming the positive control fluopyram. This study is the first to report of imidazo­[1,2-*a*]­pyridine derivatives incorporating amino acid fragments that display both nematicidal and fungicidal potential, thereby establishing a new framework for the future development of agrochemical agents.^88^

**Figure 17:**
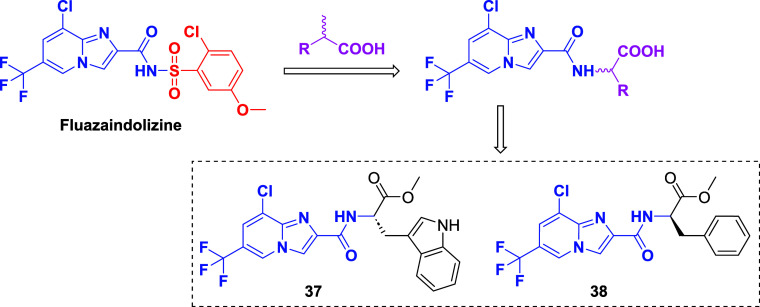
Design of compounds **37** and **38** employed a pharmacophore-guided planning approach inspired by fluazaindolizine, which couples amino acid moieties to the imidazo­[1,2-*a*]­pyridine scaffold.

### 3.3 Antitubercular

An interesting study reported the synthesis of 1,3-diaryl-substituted pyrazole-based imidazo­[1,2-*a*]­pyridine carboxamides and evaluated their antitubercular activity. Ommi and colleagues identified ten compounds with minimum inhibitory concentration (MIC) values of 0.03 μM against *Mycobacterium tuberculosis* H37Rv (Mtb H37Rv), which were deemed nontoxic to Vero cells. Compounds **39**, **40**, and **41** exhibited promising activity against drug-resistant M. tuberculosis strains and demonstrated synergistic effects with FDA-approved antituberculosis drugs (Figure 18). These compounds were 17 times more potent than streptomycin, 33 times more potent than ethambutol, and equipotent to isoniazid and rifampicin, respectively. Bacterial killing kinetic studies revealed that all three compounds exhibited bacteriostatic activity. Moreover, they showed good metabolic stability in human liver microsomes (CL_int_ ranging from 11.49 to 14.62 μL·min^–1^·mg^–1^ microsomal protein). Compounds **39**, **40**, and **41** also exhibited stable interactions with ubiquinol-cytochrome *c* reductase Cytochrome *b* subunit (QcrB) in molecular docking studies. Docking analyses indicated scores ranging from −8.669 to −9.553, whereas the cocrystallized ligand (telacebec) exhibited a binding score of −11.2 kcal·mol^–1^. Altogether, these findings highlight compounds **39**, **40**, and **41** as promising candidates for *in vivo* evaluation and the further development of new antituberculosis agents.^89^

**Figure 18:**
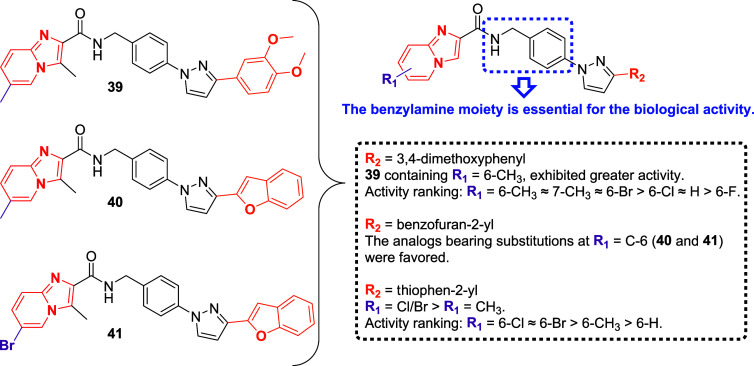
Compounds **39**-**41**, which exhibited the best antituberculosis activity, highlight the importance of substituents in the SAR.

Patel and colleagues investigated the potential of novel imidazo­[1,2-*a*]­pyridine derivatives as agents against drug-resistant *M. tuberculosis*, one of the major challenges in current tuberculosis therapy. Compound **42** exhibited notable antituberculosis activity against the H37Rv strain of *M. tuberculosis*, with an MIC value of 0.6 μM, comparable to that of the reference drug isoniazid (MIC = 0.4 μM) (Figure 19). Molecular docking studies revealed docking scores of −8.96 and −8.90 kcal·mol^–1^ for compound **42** and isoniazid, respectively. The highlighted compound displayed alkyl and π–alkyl interactions with Ala157, Leu267, Met155, and Pro156. The fluorine atom forms a halogen bond with Gln214. These findings suggest that imidazo­[1,2-*a*]­pyridine derivatives, particularly compound **42**, are promising candidates for combating drug-resistant tuberculosis. Moreover, they provide valuable insights for guiding the development of new and effective tuberculosis treatments.^90^

**Figure 19:**
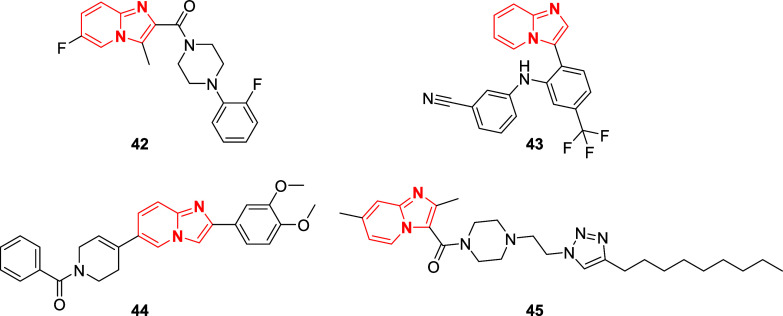
Structures of compounds **42**–**45** with antituberculosis activity.

Karale and coworkers synthesized 3-aryl-substituted imidazo­[1,2-*a*]­pyridines as potential antitubercular agents. The compounds were evaluated against *M. tuberculosis* H37Rv. The lead compound, **43**, was identified as a promising candidate for antituberculosis drug development, exhibiting a MIC value of 2.3 μg·mL^–1^ against *M. tuberculosis* H37Rv, compared to the positive control rifampicin (MIC = 0.2 μg·mL^–1^) (Figure 19). The most active derivative bearing a 3-benzonitrile substituent showed superior activity, followed by the analogs containing 6-quinoxaline and 2,4-difluorobenzene groups. The hydrogen-bond-accepting character of the aryl ring was found to enhance its biological activity. Molecular docking studies of compound **43** within the QcrB active site revealed key π–π interactions with residues Tyr389 and Trp312. Compound **43** exhibited a docking score of −9.667 kcal·mol^–1^, and the observed interactions were consistent with its superposition on telacebec. Overall, the lead compound represents an interesting candidate for further investigations.^91^

Khetmalis and colleagues, employing a molecular hybridization strategy, synthesized and evaluated a series of imidazo­[1,2-*a*]­pyridine amides. The best-performing compound, **44**, exhibited a MIC value of 0.05 μg·mL^–1^ against *M. tuberculosis* H37Rv, in contrast to the positive controls, *p*-aminosalicylic acid and ethambutol, which displayed MIC values of 12.5 and 6.25 μg·mL^–1^, respectively (Figure 19). Among the most active derivatives, selected compounds were evaluated for cytotoxicity in embryonic kidney cells to their safety in normal cells, and compound **44** was found to be nontoxic (SI > 1440). ADMET predictions indicated that compound 44 violated one of Lipinski’s rules (hydrogen bond acceptor count >5) and one of Jorgensen’s Rule of Three parameters (log S < −6.5). Molecular docking studies revealed that compound 44 interacted with residues Tyr158 and Gln100 through hydrogen bonds and formed an additional π–π interaction with Tyr158, exhibiting a glide score of −7.27 kcal·mol^–1^. Overall, the most active compound in the series demonstrated a 125-fold greater potency than the standard drug ethambutol. These findings highlight the relevance of further structural optimization and support its potential for future *in vivo* investigations.^92^

Nandikolla and coworkers designed, synthesized, and evaluated new 1,2,3-triazole analogs of imidazo­[1,2-*a*]­pyridine-3-carboxamide against *M. tuberculosis* H37Rv. The lead compound of the study, **45**, the most active derivative, exhibited MIC values of 13.74 and 24.63 μg·mL^–1^ in the microplate Alamar blue assay (MABA) and low-oxygen recovery assay (LORA), respectively (Figure 19). The positive control, isoniazid, showed MIC values of 0.36 and 76.80 μg·mL^–1^. In terms of SAR, compounds bearing *para*-substituted electron-donating groups exhibited the best activity, and derivatives with long alkyl chains or a cyclopropyl group were the most active. The *in silico* ADMET parameters were predicted, and compound **45** did not violate any of Lipinski’s Rule of Five or Rule of Three. The toxicity profile indicated that the lead compound would not be topical; overall, it exhibited drug-like properties.^93^

### 3.4 Antikinetoplastid

Agarwal and colleagues designed and synthesized imidazo­[1,2-*a*]­pyridine-chalcone conjugates as antikinetoplastid agents. The compounds were evaluated against *Trypanosoma cruzi*, *Trypanosoma brucei brucei*, *Trypanosoma brucei rhodesiense*, and *Leishmania infantum*, and their cytotoxic activity was tested against the MRC-5 cell line. Compound **47** exhibited the best activity against *T. cruzi*, with an IC_50_ value of 8.5 μM. Against *T. b. rhodesiense*, compound **46** exhibited the best activity, with an IC_50_ value of 1.13 μM. Compound **48** exhibited the most potent activity against *T. b. brucei*, with an IC_50_ value of 1.35 μM (Table 4). Among the three standout compounds, only compound 47 exhibited antikinetoplastid activity against all tested organisms. None of the compounds exhibited cytotoxicity against the MRC-5 cell line, with IC_50_ values exceeding 50 μM. Structurally, the compounds differ only by the chalcone aldehyde-derived linker, with substitutions of *p*-bromo, *o*-chloro, and *m*-chloro. None of the compounds exhibited better activity than the positive controls, benznidazole and suramin. However, the findings are promising the improvement of the structures, for potential *in vivo* studies, and development of new antikinetoplastid agents.^94^

**Table 4:**
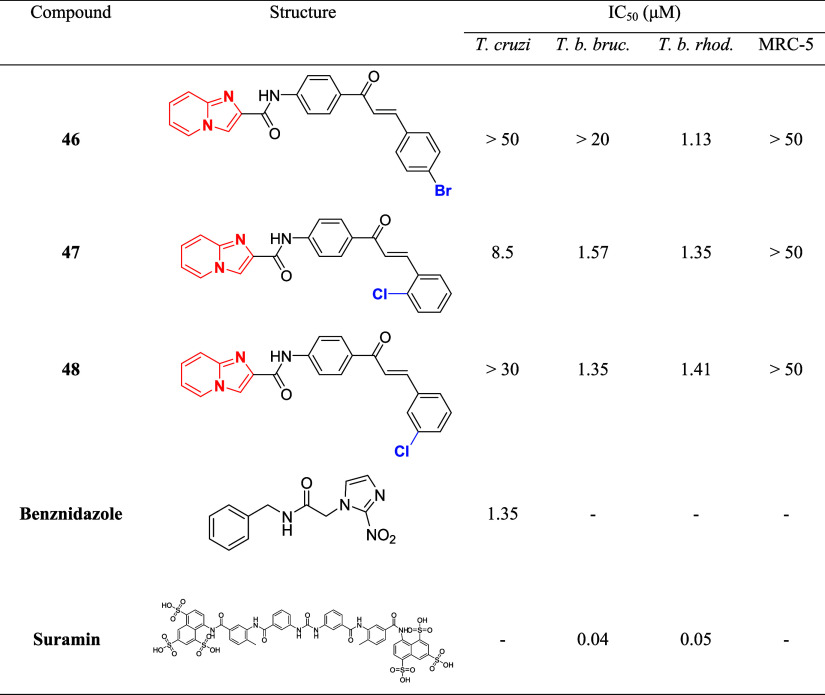
Structures of Compounds **46**–**48** Were Compared with Those of the Positive Controls Benznidazole and Suramin, along with Their Corresponding IC_50_ Values

Paoli-Lombardo and collaborators conducted a study focused on the 2- and 8-positions of the imidazo­[1,2-*a*]­pyridine ring, synthesizing 22 new derivatives. Following initial screening against the promastigote and axenic amastigote stages of *Leishmania donovani* and *Leishmania infantum*, the most promising compounds were further evaluated against the intracellular amastigote stage of *L. infantum* and assessed for their physicochemical and *in vitro* pharmacokinetic properties, leading to the identification of a novel antileishmanial candidate. The lead compound, **49**, exhibited low cytotoxicity toward HepG2 and THP-1 (human monocytic) cell lines, with CC_50_ (half-maximal cytotoxic concentration) values greater than 100 μM (Figure 20). In line with this favorable safety profile, compound **49** exhibited potent activity against intracellular amastigotes of *L. infantum*, with an EC_50_ value of 3.7 μM, compared to the positive controls miltefosine and fexinidazole, which exhibited EC_50_ values of 0.4 and 15.9 μM, respectively. Moreover, compared to previously reported derivatives (hits A and B), compound **49** displayed significantly improved aqueous solubility, good microsomal stability in mice (*t*_1/2_ > 40 min), and high gastrointestinal permeability in the PAMPA model. Collectively, these findings highlight compound **49** as an attractive candidate for further *in vivo* evaluation.^95^

**Figure 20:**
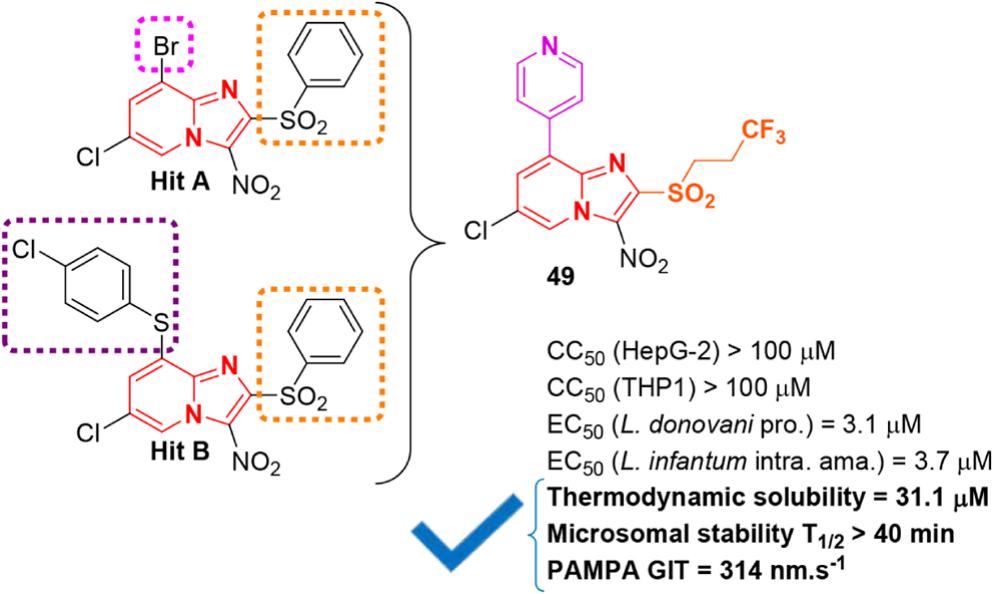
Compound **49** showed improved aqueous solubility and enhanced *in vitro* pharmacokinetic properties of the 3-nitroimidazo­[1,2-*a*]­pyridine pharmacophore compared to hits **A** and **B**.

### 3.5 Other Potential Pharmacological Applications

Kuzu and coworkers investigated cholinesterase inhibition by designing and synthesizing a series of imidazo­[1,2-*a*]­pyridine-based Mannich bases. *In vitro* studies were conducted to evaluate the inhibitory activities of the newly synthesized compounds against acetylcholinesterase (AChE) and butyrylcholinesterase (BuChE), along with enzyme kinetics analyses. The lead compound of this study, **50**, exhibited the most promising inhibitory activity, with IC_50_ values of 57.75 and 99.0 nM against AChE and BuChE, respectively (Figure 21). The positive controls, tacrine and donepezil, showed IC_50_ values of 243.11 and 192.56 nM, and 69.30 and 63.00 nM for AChE and BuChE, respectively. SAR analysis revealed the following decreasing order of AChE inhibitory activity (IC_50_, nM) for substituents at the C-2 position of the imidazo­[1,2-*a*]­pyridine scaffold: **50** (naphthyl) > donepezil ≫ p-trichloromethyl > [1,1′-biphenyl]-4-yl >4-methylphenyl >4-hydroxyphenyl >4-nitrophenyl ≫ 200 nM (remaining compounds). In contrast, the decreasing order of BuChE inhibitory activity (IC_50_, nM) for the same substitution pattern was donepezil > **50** (naphthyl) > p-trichloromethyl > [1,1′-biphenyl]-4-yl ≫ 200 nM (remaining compounds). Molecular docking studies using the crystal structures of human AChE (*h*AChE) and human BuChE (*h*BuChE) revealed that compound **50** exhibited a high binding affinity, targeting both the catalytic anionic site (CAS) and the peripheral anionic site (PAS). For AChE, donepezil displayed a docking score of −11.36 kcal·mol^–1^, whereas compound **50** showed a more favorable score of −11.79 kcal·mol^–1^; notably, both compounds formed hydrogen bonds with the residue Phe295. For BuChE, donepezil exhibited a docking score of −8.90 kcal·mol^–1^, whereas compound **50** achieved a stronger interaction with a docking score of −9.35 kcal·mol^–1^. Furthermore, pharmacokinetic profiling and preliminary ADMET evaluations indicated that compound **50** possessed more favorable drug-like properties. Collectively, these results highlight a new milestone underscoring the relevance of the imidazo­[1,2-*a*]­pyridine scaffold for the future development of therapeutic agents targeting Alzheimer’s disease.^96^

**Figure 21:**
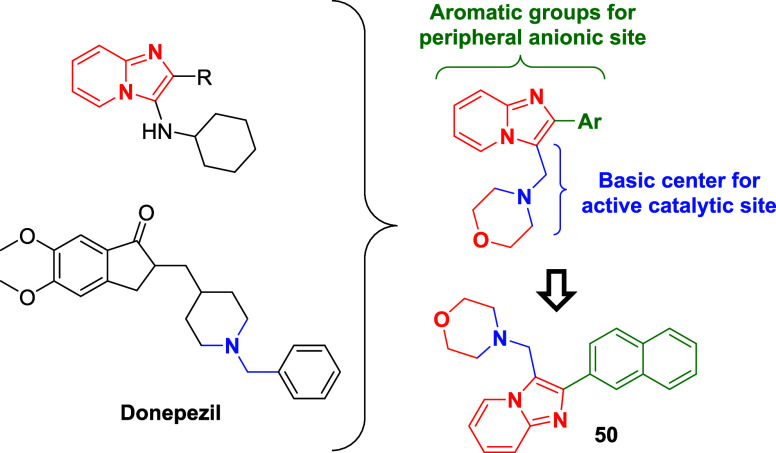
Compounds were designed based on the imidazo­[1,2-*a*]­pyridine scaffold and the drug donepezil, leading to the study’s lead compound, **50**.

Ahmadi and colleagues designed and synthesized novel imidazo­[1,2-*a*]­pyridine derivatives using rational drug design strategies, with the specific aim of developing new potential COX-2 inhibitors. Accordingly, this study aimed to contribute to the development of selective COX-2 inhibitors with improved therapeutic benefits. Overall, the results demonstrated that most of the synthesized compounds exhibited inhibitory activity with marked selectivity for COX-2. The lead compound, **51**, displayed an excellent SI of 252 for the COX-1/COX-2 ratio, with IC_50_ values of 12.6 and 0.05 μM against COX-1 and COX-2, respectively (Figure 22). The positive control used in the study, celecoxib, exhibited an SI of 90 and IC_50_ values of 24.3 and 0.07 μM against COX-1 and COX-2, respectively. *In vivo* analgesic activity assays of the most potent COX-2 inhibitors revealed that compound **51** produced the most pronounced effect, with a median effective dose (ED_50_) value of 12.35 mg·kg^–1^. Overall, the majority of the tested compounds demonstrated both high potency and selectivity for COX-2 inhibition. Furthermore, the observed analgesic effects of the most active compounds were attributed to the inhibition of cyclooxygenase activity. Collectively, the diaryl imidazo­[1,2-*a*]­pyridine scaffold exhibited a high potential for selective COX-2 inhibition, reinforcing its relevance for the development of novel anti-inflammatory and analgesic agents.^97^

**Figure 22:**
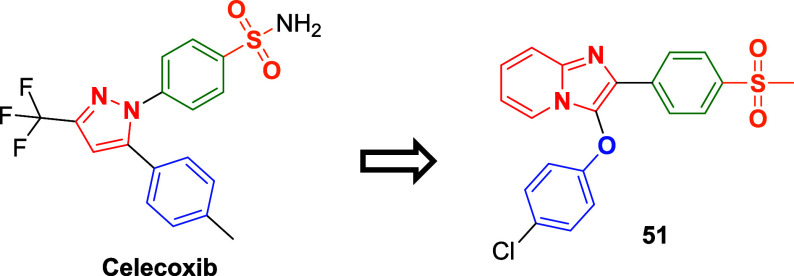
Structure of lead compound **51** compared to celecoxib. The structural modification from a 4-sulfonamide to a 4-methylsulfonylphenyl group enhances the COX-2 inhibitory activity.

Padmaja and colleagues designed and synthesized novel pyridine-linked imidazo­[1,2-*a*]­pyridine derivatives and evaluated their *in vitro* α-glucosidase inhibitory and antioxidant activities. The compounds were assessed for α-glucosidase inhibition, and lead compound **52** exhibited the highest potency, with an IC_50_ value of 3.7 μM, approximately 18-fold more potent than acarbose, the reference inhibitor (IC_50_ = 67.4 μM) (Figure 23). The increasing order of α-glucosidase inhibitory activity was as follows: compounds with IC_50_ > 30 μM < 3,4,5-trimethoxyphenyl <3,5-difluorophenyl <2,4,6-trimethylphenyl <3-(trifluoromethyl)­phenyl <2-naphthyl (**52**). Regarding antioxidant activity, compound **52** again proved to be the most potent, exhibiting an IC_50_ value of 2.496 μM, compared with ascorbic acid (IC_50_ = 87.814 μM) in the DPPH (2,2-diphenyl-1-picrylhydrazyl) radical scavenging assay. Overall, most of the synthesized compounds demonstrated excellent antioxidant properties relative to the positive control, ascorbic acid. Molecular docking studies revealed a glide score of −9.45 kcal·mol^–1^ for compound **52**, with a hydrogen bond interaction involving Asp420, whereas acarbose showed a glide score of −14.78 kcal·mol^–1^, forming hydrogen bonds with Arg404, Asp420, Glu481, Asn197, His478, Asp73, and Asp307. Finally, the *in silico* physicochemical and pharmacokinetic properties of the synthesized compounds were within acceptable ranges, supporting their drug-likeness potential.^98^

**Figure 23:**
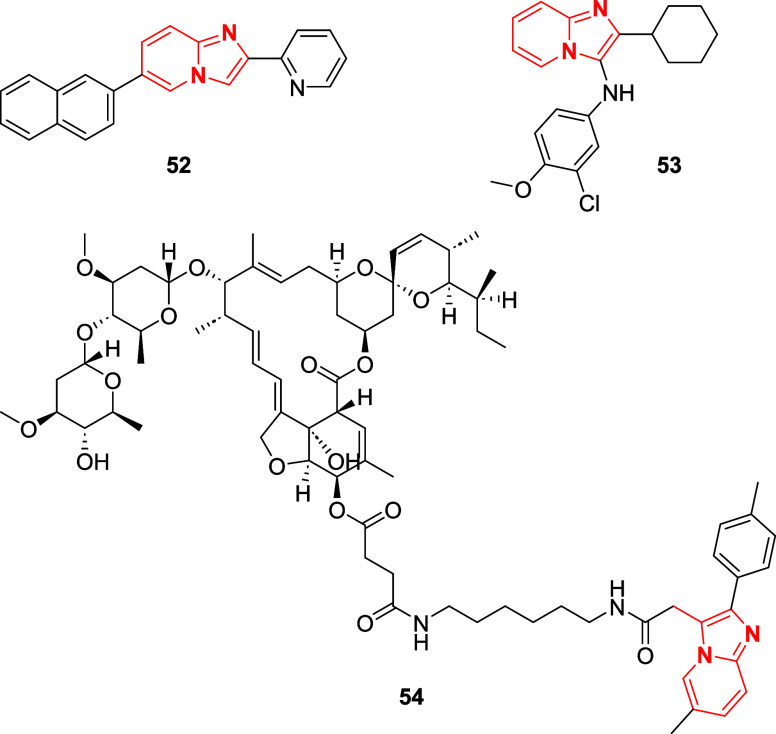
Structures of compounds **52**–**54** exhibiting distinct biological activities.

Wang and collaborators identified imidazo­[1,2-*a*]­pyridin-3-amine as a pharmacologically relevant scaffold for effective *in vivo* inhibition of ferroptosis. This study adopted a scaffold repurposing strategy, repositioning this chemotype as a novel class of ferroptosis inhibitors for oxidative stress-related diseases. The lead compound, **53**, exhibited a high free radical scavenging capacity and effective membrane retention, resulting in pronounced suppression of lipid peroxidation and strong inhibitory activity against cellular ferroptosis at nanomolar concentrations (Figure 23). Compound **53** exhibited an IC_50_ of 97 nM, compared to 20 nM for ferrostatin-1, the first synthetic ferroptosis inhibitor. SAR studies have revealed that substituents at the C-6 and C-7 positions are favorable for activity, whereas substitution at C-8 is detrimental. Lipophilic cyclohexyl groups and low-polarity phenyl substituents further enhanced the activity. Importantly, SAR analysis established that the 3-amino group is essential for antiferroptotic activity. In addition, the lead compound exhibited low cytotoxicity, high metabolic stability, and favorable predicted blood–brain barrier permeability, culminating in pronounced *in vitro* neuroprotection against ischemic brain injury in mice. Overall, this study is particularly valuable from a drug repositioning perspective, as it not only identifies potent ferroptosis inhibitors but also introduces structurally innovative features that expand the chemical space for the discovery of next-generation antiferroptotic agents.^99^

Volkova and coworkers synthesized and evaluated avermectin-imidazo­[1,2-*a*]­pyridine hybrids as potential GABA_*A*_ receptor modulators. A series of hybrids combining avermectin and imidazo­[1,2-*a*]­pyridine pharmacophores was designed and synthesized to act as positive allosteric modulators of GABA_*A*_ receptors. Lead compounds were identified using radioligand competition assays. *In vitro* experiments showed that the lead compound, **54**, exhibited high potency, with an IC_50_ of 207 nM, compared to the positive control zolpidem (IC_50_ = 43.3 nM) for binding to the benzodiazepine site of GABA_*A*_ receptors (Figure 23). The functional properties of compound **54**, which exhibited the highest affinity as a positive allosteric modulator of GABA_*A*_ receptors, were further evaluated using patch-clamp electrophysiological recordings of GABA-mediated currents in rat cerebellar Purkinje neurons. The biological efficacy of the hybrid molecules (avermectin-imidazo­[1,2-*a*]­pyridine) was demonstrated *in vivo* using behavioral assays in adult zebrafish. Overall, the investigation of these hybrids as GABA_*A*_ receptor modulators is highly relevant for the discovery of novel, safe, and effective therapeutics for treating neurological disorders.^100^

## 4 Conclusion

The imidazo­[1,2-*a]*pyridine scaffold is a highly relevant and versatile platform in medicinal chemistry. Recent advances in innovative and sustainable synthetic methods and their expanding application across multiple therapeutic areas demonstrate that imidazo­[1,2-*a*]­pyridine continues to stand as a bona fide privileged scaffold with considerable potential to contribute to the development of new bioactive entities. Although the *in vitro* activity and the breadth of applications are promising, significant challenges persist: many compounds have not progressed to preclinical evaluation, substantial gaps remain in ADME/Tox data, and optimization toward drug-like properties still requires systematic efforts. Therefore, an updated and comprehensive systematic review, such as the present one, is fully justified to consolidate recent findings, identify patterns and knowledge gaps, and guide future research and development efforts using imidazo­[1,2-*a*]­pyridine as the central core.

Despite the progress achieved, this review underscores that the field is far from being saturated. Conversely, recent methodological developments, including multicomponent reactions, modernized cycloadditions, selective C–H functionalization, and sustainable biomass-derived routes, have significantly expanded the accessible chemical space for the synthesis of imidazo­[1,2-*a*]­pyridine derivatives. These innovations allow not only the rapid generation of diversified libraries but also the incorporation of unprecedented functionalities that enable previously unattainable molecular interactions. From a biological standpoint, a robust expansion toward new therapeutic areas has emerged, including kinase inhibition, neuroenzyme modulation, antiparasitic, and anti-inflammatory activities, highlighting that imidazo­[1,2-*a*]­pyridines continue to offer promising solutions to complex biomedical challenges.

However, important gaps remain. The structural diversity explored to date is still largely confined to specific substitution patterns, particularly at C-2 and C-3, and through simple modifications of the fused ring. More ambitious strategies involving deep bioisosterism, dual-scaffold hybrids, and polar or fragment-like modulators remain significantly underexplored. Although many compounds exhibit potent *in vitro* activity, *in vivo* studies and pharmacokinetic profiles that support a clear preclinical development trajectory are often lacking. In many cases, potency is established, but essential attributes such as metabolic stability, selectivity, permeability, toxicity, and solubility do not advance accordingly, each of which is critical for converting hits into viable leads.

Considering all the above factors into account, the future of imidazo­[1,2-*a*]­pyridine derivatives look very promising. From a synthetic viewpoint, opportunities still remain to investigate greener methodologies, earth-abundant catalysts, advanced photocatalysis, electrosynthesis and modular approaches that minimize steps and waste in the core structural diversification. Structurally, the extension of the range of unconventional substitution patterns on the imidazo­[1,2-*a*]­pyridine nucleus and the implementation of strategies of fragment-based drug design may open up previously inaccessible pathways. Pharmacologically, it is expected to be a natural move from only in vitro testing to thorough studies of these derivatives, including animal models, pharmacokinetic properties and safety optimization. Modern computational tools, machine learning, large scale virtual screening and multidimensional similarity analyses can greatly accelerate the identification of promising imidazo­[1,2-*a*]­pyridine candidates.

Taken together, imidazo­[1,2-*a*]­pyridine derivatives remain among the most fertile, resilient, and adaptable scaffolds for modern pharmaceutical innovations. The convergence of synthetic advances, biological expansion, molecular diversification, and the integration of emerging technologies positions this core as a central component in the next generation of drug discovery efforts. However, to fully realize the potential of the imidazo­[1,2-*a*]­pyridine scaffold, it is essential to overcome critical gaps, particularly those related to ADME/Tox and more daring structural exploration. If these challenges are approached with rigor and creativity, the imidazo­[1,2-*a*]­pyridine scaffold will continue to play a strategic role in building innovative molecules capable of addressing current and future biomedical needs.

## Data Availability

The authors confirm that the data supporting the findings of this study are available within the article. Further inquiries can be directed to the corresponding author.
